# The acyl-CoA synthetase *Tg*ACS1 allows neutral lipid metabolism and extracellular motility in *Toxoplasma gondii* through relocation via its peroxisomal targeting sequence (PTS) under low nutrient conditions

**DOI:** 10.1128/mbio.00427-24

**Published:** 2024-03-19

**Authors:** Sarah Charital, Serena Shunmugam, Sheena Dass, Anna Maria Alazzi, Christophe-Sébastien Arnold, Nicholas J. Katris, Samuel Duley, Nyamekye A. Quansah, Fabien Pierrel, Jérôme Govin, Yoshiki Yamaryo-Botté, Cyrille Y. Botté

**Affiliations:** 1 Apicolipid Team, Institute for Advanced Biosciences, CNRS UMR5309, INSERM U1209, Université Grenoble Alpes, Grenoble, France; 2 Team Govin, Institute for Advanced Biosciences, CNRS UMR5309, INSERM U1209, Université Grenoble Alpes, Grenoble, France; 3 Université Grenoble Alpes, CNRS, Grenoble INP, TIMC-IMAG, Grenoble, France; UT Southwestern Medical Center, Dallas, Texas, USA

**Keywords:** *Toxoplasma gondii*, lipid, fatty acid, acylCoA synthetase, lipidomics, fatty acid metabolism, motility

## Abstract

Apicomplexa parasites cause major diseases such as toxoplasmosis and malaria that have major health and economic burdens. These unicellular pathogens are obligate intracellular parasites that heavily depend on lipid metabolism for the survival within their hosts. Their lipid synthesis relies on an essential combination of fatty acids (FAs) obtained from both *de novo* synthesis and scavenging from the host. The constant flux of scavenged FA needs to be channeled toward parasite lipid storage, and these FA storages are timely mobilized during parasite division. In eukaryotes, the utilization of FA relies on their obligate metabolic activation mediated by acyl-co-enzyme A (CoA) synthases (ACSs), which catalyze the thioesterification of FA to a CoA. Besides the essential functions of FA for parasite survival, the presence and roles of ACS are yet to be determined in Apicomplexa. Here, we identified *Tg*ACS1 as a *Toxoplasma gondii* cytosolic ACS that is involved in FA mobilization in the parasite specifically during low host nutrient conditions, especially in extracellular stages where it adopts a different localization. Heterologous complementation of yeast ACS mutants confirmed *Tg*ACS1 as being an Acyl-CoA synthetase of the bubble gum family that is most likely involved in β-oxidation processes. We further demonstrate that *Tg*ACS1 is critical for gliding motility of extracellular parasite facing low nutrient conditions, by relocating to peroxisomal-like area.

**IMPORTANCE:**

*Toxoplasma gondii*, causing human toxoplasmosis, is an Apicomplexa parasite and model within this phylum that hosts major infectious agents, such as *Plasmodium* spp., responsible for malaria. The diseases caused by apicomplexans are responsible for major social and economic burdens affecting hundreds of millions of people, like toxoplasmosis chronically present in about one-third of the world’s population. Lack of efficient vaccines, rapid emergence of resistance to existing treatments, and toxic side effects of current treatments all argue for the urgent need to develop new therapeutic tools to combat these diseases. Understanding the key metabolic pathways sustaining host-intracellular parasite interactions is pivotal to develop new efficient ways to kill these parasites. Current consensus supports parasite lipid synthesis and trafficking as pertinent target for novel treatments. Many processes of this essential lipid metabolism in the parasite are not fully understood. The capacity for the parasites to sense and metabolically adapt to the host physiological conditions has only recently been unraveled. Our results clearly indicate the role of acyl-co-enzyme A (CoA) synthetases for the essential metabolic activation of fatty acid (FA) used to maintain parasite propagation and survival. The significance of our research is (i) the identification of seven of these enzymes that localize at different cellular areas in *T. gondii* parasites; (ii) using lipidomic approaches, we show that *Tg*ACS1 mobilizes FA under low host nutrient content; (iii) yeast complementation showed that acyl-CoA synthase 1 (ACS1) is an ACS that is likely involved in peroxisomal β-oxidation; (iv) the importance of the peroxisomal targeting sequence for correct localization of *Tg*ACS1 to a peroxisomal-like compartment in extracellular parasites; and lastly, (v) that *Tg*ACS1 has a crucial role in energy production and extracellular parasite motility.

## INTRODUCTION

Apicomplexa parasites are a group of unicellular eukaryotes that include obligate intracellular parasites responsible for major human and cattle diseases. These include *Toxoplasma gondii*, *Plasmodium* sp., and *Cryptosporidium parvum,* which are the causing agents of the infectious diseases toxoplasmosis, malaria, and cryptosporidiosis, respectively. Eradicating these diseases is a worldwide priority, and the renewal of our therapeutic arsenal relies on understanding host-parasite interactions sustaining parasite survival. Specific metabolic pathways allowing nutrient acquisition, intracellular development, and parasite propagation represent ideal targets for drug development. Lipid synthesis, acquisition, and homeostasis have been shown as key metabolic pathways for parasite survival and pathogenicity (1, 2).

During the intracellular development of *T. gondii*, lipids play an essential role (i) by providing structural building blocks for membrane biogenesis (3, 4), (ii) as signaling molecules participating in key events like invasion and egress through microneme secretion (5–7), and (iii) as stored fuels, which play a critical role to maintain proper intracellular development (8, 9). The propagation and survival of *T. gondii* thus rely on the acquisition of large and continuous amounts of lipids. To cope with this continuous need for lipids within the different host cells environments, *T. gondii* has evolved complex and sometimes redundant metabolic pathways to acquire its lipid, and, more particularly, their key hydrophobic building blocks, fatty acids (FAs). Those FAs are obtained by an essential combination of (i) adaptable *de novo* syntheses/remodeling within the parasite organelles and (ii) massive scavenging from the host cell and the host cell environment. The *de novo* synthesis of FA in the parasite mainly relies on the metabolically flexible production by the apicoplast prokaryotic type II fatty acid synthesis (FASII) pathway and the Endoplasmic Reticulum (ER)-based FA elongation/desaturation pathway (4, 10–14). At the same time, the parasite constantly and massively scavenges lipids and FA from the host (15)(4, 8, 9, 12, 16–18). The current consensus on parasite lipid metabolism suggests that both FA sources from the apicoplast FASII, and scavenged from the host, are critical for intracellular parasite viability. Indeed, both FA sources are normally combined to generate “patchwork phospholipid molecules” making the bulk of *T. gondii* lipid composition (3).

In addition, the constant flux of scavenged FA needs to be channeled toward the parasite’s lipid storage, i.e., lipid droplets (LDs), through the action of a pivotal phosphatidic acid phosphatase, *Tg*Lipin (9). This allows for the circumvention of a lipotoxic effect due to the lethal accumulation of FA in the parasite and a timely mobilization of FA when the parasite needs it, to constitutively allow parasite division, and/or in response to atypical/starvation conditions.

The FA flux balance is further controlled by the change in the metabolic input for both FA sources (*de novo* vs scavenged), which is regulated by host nutrient availability (12, 13). Indeed, *Toxoplasma gondii* is capable of infecting virtually any nucleated cell from warm-blooded animals, thereby reflecting its metabolic plasticity, giving it the capacities to adapt to different nutritional environments. Under high host lipid content, FASII can be downregulated and maintained at a basal level, or even become dispensable, as the parasite relies principally on host scavenging (12–14). Under low host lipid content, however, the FASII activity is significantly upregulated concomitantly with an upregulation of the scavenging of host membrane material to maintain the parasite’s demand for lipids (12, 19). The availability of FAs from the host and exogenous environment, as well as their utilization/mobilization for a targeted localization is thus a critical function to maintain for the parasite. However, very little is known about the molecular actors allowing FA acquisition/manipulation in the parasite.

**Fig 1:**
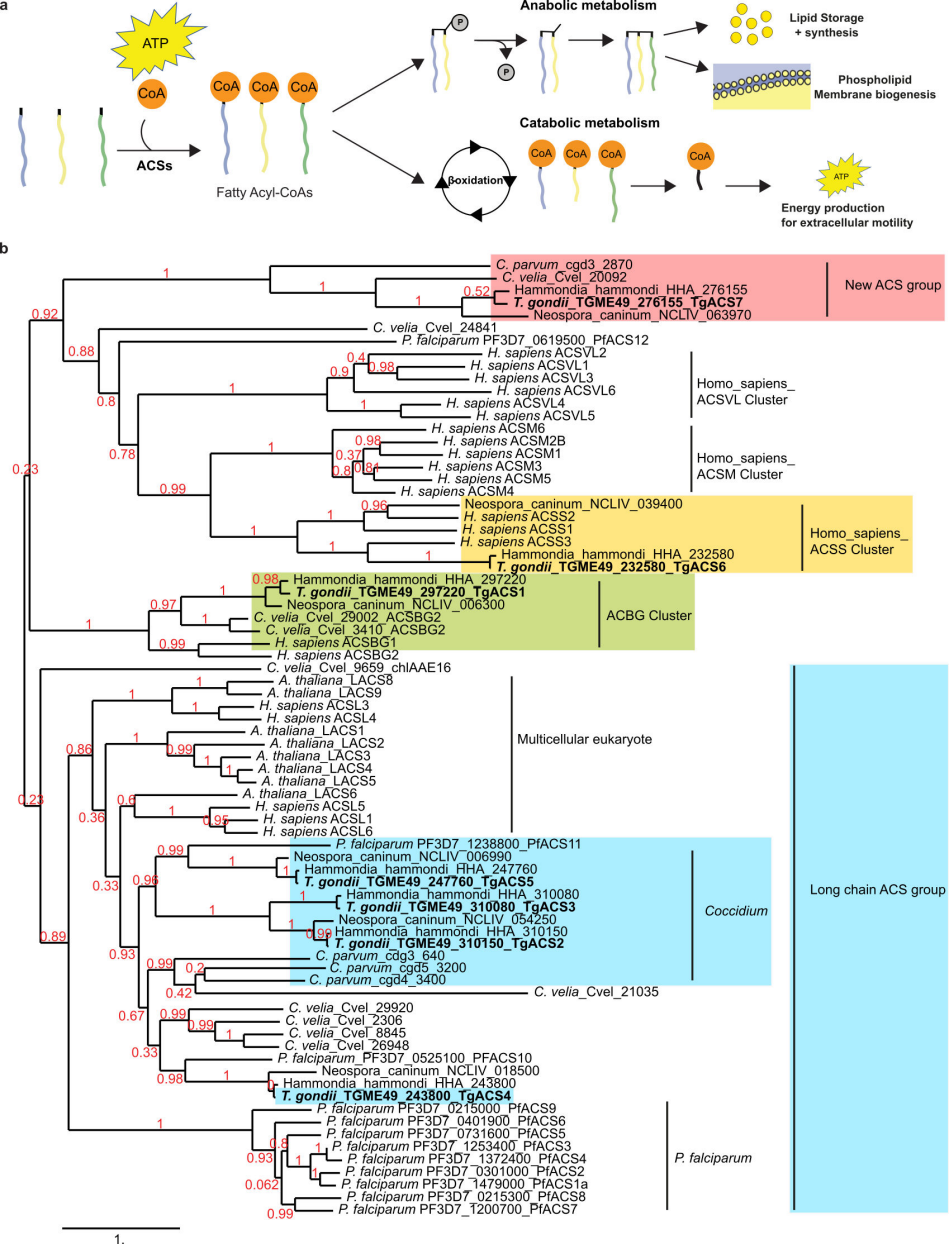
Identification of seven acyl-CoA synthetase in *T. gondii* and their cellular localizations. (**a**) Anabolic and catabolic processes resulting from the thioesterification of a fatty acid by an ACS using ATP. (**b**) Phylogenetic analyses of all seven ACS found in the genome of *Toxoplasma gondii* parasites. All seven *Tg*ACSs appear in specified clusters based on their homology with their eukaryotic counterparts: *Tg*ACS1 in ACSBG cluster; *Tg*ACS2, *Tg*ACS3, *Tg*ACS4, and *Tg*ACS5 in the long-chain ACS group; *Tg*ACS6 in the short-chain ACS cluster; *Tg*ACS7 in a separate cluster specific to Coccidia and Chromerida. The red number represents the value of each bootstrap. This phylogenetic tree was generated online using Phylogeny.fr.

One of the key biochemical steps required for general FA utilization and trafficking is their obligate metabolic activation by thioesterification to a co-enzyme A (CoA). This metabolic activation step is catalyzed by the essential acyl-CoA synthetase (ACS or acyl-CoA ligase, EC 6.2.1.3) family (20). Biochemically, ACS catalyzes a two-step reaction to form Acyl-CoAs (Fig. 1a). The initial step involves the formation of an adenylated intermediate through the hydrolysis of an ATP molecule, thus releasing pyrophosphate (21). The ATP-activated enzyme then binds to the carboxyl group of an incoming free FA (FFA) through an acyl bond to the phosphoryl group of AMP. The final acyl-CoA product is formed after the transfer of the acyl group to the sulfhydryl group of coenzyme A, thereby releasing AMP [(21), Fig. S1].

ACSs can be grouped according to their substrate. In humans, there are 26 ACSs and 11 of which are long-chain (ACSLs) and very long-chain ACSs (ACSVLs) (21). These ACSLs and ACSVLs specifically activate fatty acids that are 16 to 22 carbons in length (20). The ACSLs, however, typically activate highly abundant fatty acids in nature such as palmitate (C16:0) and oleate (C18:1). Other ACSs include short-chain ACSs that typically activate acetate, propionate, or butyrate, and medium-chain ACSs (ACSMs), and that activate fatty acids with chain lengths ranging from 6 to 10 carbons (20). Lastly, the bubble gum ACSs gene family (or ACSBG family also known as lipidosin) can activate FAs with 16 to 24 carbons chains usually to fuel belowβ-oxidation, as described (22).

ACSs are indispensable for the metabolism of acyl-CoAs. Their function depends on many criteria, which can be substrate specificity (based on FA chain length and degree of unsaturation), cellular localization, expression patterns, interaction partners, and more. They are usually involved in (i) membrane biogenesis by providing acyl-CoAs for phospholipids/neutral/storage lipids synthesis and potential FA modifications (elongation, desaturation, recycling), (ii) for protein/sterol acylation, or (iii) when located in catabolic organelles such as mitochondria or peroxisomes, allow the FA degradation by a process known as β-oxidation, which can fuel the TriCarboxylic Acid (TCA) cycle for energy purposes (20–26). For example, the human prostate ACSL1, for example, localized at mitochondria, is directly linked to the FA β-oxidation function (27,28, 29). ACSL3, which has been found on LDs and ER, participates in FA uptake and glycerolipid biosynthesis within the endomembrane system (30). More importantly, bubble gum ACSs, ACSBG, are specific to a single target organelle, peroxisome, where β-oxidation takes place in certain organisms. Peroxisomal ACSBG cannot complement the mitochondrial ACSs and vice versa (31, 32).

Peroxisome is a catabolic organelle often associated with the β-oxidation. Most of the peroxisome-localized proteins have specific targeted signal peptides (Peroxisomal Targeting Sequence or PTS). The presence of PTS in *T. gondii* brought the debate in the very existence/presence of peroxisomes in Apicomplexa parasites. Its existence has long been debated, due to the initial discoveries and localization(s) of catalase, and the presence of putative typical peroxisomal targeting sequence (PTS) in many proteins encoded in the parasite genome (33, 34). Indeed, catalase, a typical marker enzyme for peroxisomes in eukaryotic cells, was initially identified in *T. gondii* and localized to a unique spot above the nucleus and below the apicoplast, close to the Golgi apparatus. However, a further study using (i) antibodies raised against catalase and (ii) a Green Fluorescent Protein (GFP) constructs fused to predicted PTS revealed ambiguous dual cytosol localizations, although a similar apical-Golgi-like spot was initially reported (34). Biochemical separations-fractionations from the same study concluded to a possible dual cytosolic and organellar localization of catalase in *T. gondii* tachyzoites, but somehow dismissed the putative presence of typical peroxisomes in *T. gondii*. A few years later, Lige et al. (35) identified a *T. gondii* homolog of the lipid transporter Sterol Carrier Protein 2 (SCP2), usually enabling β-oxidation in eukaryotic peroxisome, *Tg*SCP2 (35, 36). *Tg*SCP2 carries a putative C-terminal PTS1, which is sufficient to allow the import of a GFP fusion into yeast peroxisome, and enables to visualize a dual localization for *Tg*SCP2: a broad cytosolic one and more punctate-condensed spots in the cytosol of *T. gondii* tachyzoites (35). Furthermore, eukaryotic peroxisome biogenesis and protein import typically require proteins called peroxins or PEX. Very interestingly, two studies independently identified strong homologs of PEX in *T. gondii*, notably *Tg*PEX5, all bearing putative PTS and most likely allowing peroxisome biogenesis and protein import. Indeed, PEX5 can recognize and bind to the proteins bearing a PTS1, allowing the transport of any soluble proteins destined to peroxisomal lumen (37, 38). It was further demonstrated that the PTS1-binding domain of *Tg*PEX5, which is usually enabling peroxisomal protein import in eukaryotes, was interacting with the PTS1 of *Tg*SCP2 *in vitro* (38). Furthermore, *Tg*PEX5 was able to rescue a PEX-KO in yeast, and the PTS1 from the yeast PEX5 was able to direct a GFP fusion to distinct cytosolic/punctate localization similar to those of *Tg*SCP2 in *T. gondii* (38). Finally, bioinformatic analyses of the PTS1 proteome in *T. gondii* revealed the presence of the complete set of enzymes putatively responsible for β-oxidation in the parasite (37). The actual biochemical nature and existence of a peroxisome is currently unclear, more particularly whether it is a “conventional” membrane-bound organelle or whether it could be membrane-less, such as a liquid-liquid phase separation (LLPS) structure. Taken together, and despite some conflicting data, there are experimental evidence for the presence of non-conventional peroxisome-like structures in *T. gondii* that could be putatively involved in lipid utilization for β-oxidation, hence energetic purposes.

In terms of energy generation, Apicomplexa parasites have the capacity to induce metabolic adaptation in response to environmental changes, and thus switch carbon sources to generate ATP and reduce power to fuel glycolysis, mitochondrial TCA cycle, and oxidative phosphorylation (2, 39, 40). It was shown that intracellular parasites mainly use host glucose, which is catabolized via parasite glycolysis and coupled to the mitochondrial TCA cycle together to generate reducing power fueled to oxidative phosphorylation to form ATP. The parasite can also utilize glutamine, at the same time or in place of glucose when deprived from it. Glutamine can be directly used as such or further converted to gamma-aminobutyric acid (GABA), which can be stored, and both can be used as carbon sources to fuel the TCA cycle (40, 41). In extracellular parasite, conditions are more scarce, yet the parasite can remain active, motile, and survive for 1 h without any exogenous source of carbon, thus pointing at their capacity to use other energy storage. Indeed, it was shown that GABA constitutes one such energy storage that fuels parasite motility in extracellular parasites (40). Another major carbon source for energy generation, and which existence has long been debated, is parasite lipid β-oxidation. Its presence and activity is currently unknown but, by definition, could use FA from lipid storage, i.e., lipid droplets, as carbon sources to generate acetyl-CoA and reducing power for ATP generation through oxidative phosphorylation. To do so, the parasite would need the full set of protein allowing the mobilization, traffic of FA, and the catabolic enzymes to perform β-oxidation. Indeed, *T. gondii* putatively possesses the complete set of encoded enzymes capable of performing β-oxidation, and they are all predicted to bear a PTS1, thus potentially operating together with other proteins bearing a predicted PTS in a putative peroxisome (37). Importantly, β-oxidation requires specific ACS, i.e., ACSBGs, to handle the FA to be catabolized. The current presence and functions of ACS in *T. gondii* are yet to be determined.

FA acquisition and metabolism are essential for parasite survival, and hence, the role of the ACSs must be critical for parasite survival. ACSs should be involved at all levels of FA acquisition/trafficking, i.e., activation of FA either derived from *de novo* synthetic machinery or from host scavenging. This is notably supported by the existence of ACS encoding genes in the genome of *Cryptosporidium* and *Theileria,* which lack the *de novo* fatty acid biosynthetic machinery (10). Furthermore, *Cryptosporidium parvum*, which lacks the apicoplast, and its FA *de novo* synthesis pathway, the FASII, have been reported to have three AMP-binding domains containing long-chain fatty acid CoA ligases, of which *Cp*ACS1 and *Cp*ACS2 have been biochemically characterized as functional ACSs (42). The localization of *Cryptosporidium parvum Cp*ACS1 at the apical end of cell-free sporozoites has been linked to FA biosynthesis required during invasion, and/or early-stage development (43). *Plasmodium falciparum*, on the other hand, has a large family of ACSs encoded by 13 genes (*Pf*ACS1a*–Pf*ACS12), whose functions remain to be determined (44). Interestingly, *P. falciparum* ACSs are also the only family of metabolic enzymes that are encoded by genes spanning into the sub-telomeric region, which usually harbors the parasite’s virulence genes and favors gene duplication, thus pointing at the importance of ACS for *Plasmodium* (44). Drugs specific to ACSs in *C. parvum* and *P. falciparum* have shown potent parasite-killing activity, thereby hinting that these enzymatic reactions are potential Achilles heels for apicomplexans (43, 45). The existence of several ACSs in the *Plasmodium* (and likely in *Toxoplasma* genomes) is indicative of their unique and central functions for maintaining parasite survival. These parasites harbor extensive and diverse ranges of FA, varying in chain lengths, degree of saturation/unsaturation, and, more importantly, a very complex cycle of importing/recycling/fluxing of said FAs. It is therefore not surprising that the parasites have a wider range of ACS encoding genes, to putatively maintain such an important function for the parasite and/or to deal with the many FAs needed by the parasite. Although the presence of an ACS was reported in *T. gondii* by bioinformatics analysis (37), the number of ACS and their functions in *T. gondii* are still unknown.

Here, we report that *T. gondii* encodes and harbors seven non-redundant putative ACSs. We demonstrate that the one we named *Tg*ACS1 has a specific role for FA activation and mobilization when parasites face low nutrient environments and, more specifically, during extracellular stages. When the parasite egresses the host cell in the search of new host cells to invade, *Tg*ACS1 is re-localized from the cytosol when intracellular to a different apical locus, thanks to its type 1 peroxisomal targeting sequence (PTS1). *Tg*ACS1 disruption deeply affects extracellular parasite gliding motility, especially in low nutrient conditions. Moreover, we found that *Tg*ACS1 belongs to the ACS bubble gum family and can only complement the yeast Knock-Out (KO) mutant strain lacking a bubble gum ACS specific for β-oxidation but no other yeast mutant lacking regular ACS. Therefore, this strongly suggests that *Tg*ACS1 is involved in providing energy through FA mobilization, potentially to fuel β-oxidation in a peroxisomal-like area of the parasite, this under low nutrient content.

## RESULTS

### Identification of seven genes encoding putative ACS enzymes within the *Toxoplasma* genome

To identify putative members of the ACS gene family in *Toxoplasma gondii*, we searched for genes bearing the typical AMP-binding domain found in eukaryotic ACSs. By mining the ToxoDB database (http://toxodb.org/toxo/), we were able to identify several candidate genes. Within these candidates bearing the AMP-binding domain, seven proteins were identified bearing highly conserved signature ACS motifs, named motif I–V (16). We assigned the seven putative *T. gondii* ACS (*Tg*ACS) candidates as *Tg*ACS1 (TGGT1_297220), *Tg*ACS2 (TGGT1_310150), *Tg*ACS3 (TGGT1_310080), *Tg*ACS4 (TGGT1_243800), *Tg*ACS5 (TGGT1_247760), *Tg*ACS6 (TGGT1_232580), and *Tg*ACS7 (TGGT1_276155) (Fig. 1b). Phylogenetic analysis allowed the regrouping of the *Tg*ACS candidates into specific clusters based on their homology to other eukaryotic counterparts (Fig. 1b). *Tg*ACS1 clustered with the human ACSs belonging to the bubble gum gene family (ACSBG), also known to be involved in β-oxidation, i.e., FA catabolic pathway allowing the generation of reducing power and energy to the cell (25). Using bioinformatic approaches, *Tg*ACS1 has previously been predicted to be involved in a putative peroxisomal β-oxidation pathway, due to the presence of a putative peroxisome targeting sequence, PTS1, and its known function for β-oxidation in other models (37). Taking into account the CRISPR (Clustered Regularly Interspaced Short Palindromic Repeats)-based phenotype scores of *Tg*ACSs, which depict their putative importance/essentiality for intracellular development (46), and the predicted function of *Tg*ACS1 in link with the use of FA for energetic purposes, we decided to focus on *Tg*ACS1.

### *Tg*ACS1 is a conserved bubble gum acyl-CoA synthetase present in cytosolic punctates bearing a peroxisomal targeting sequence

**Fig 2:**
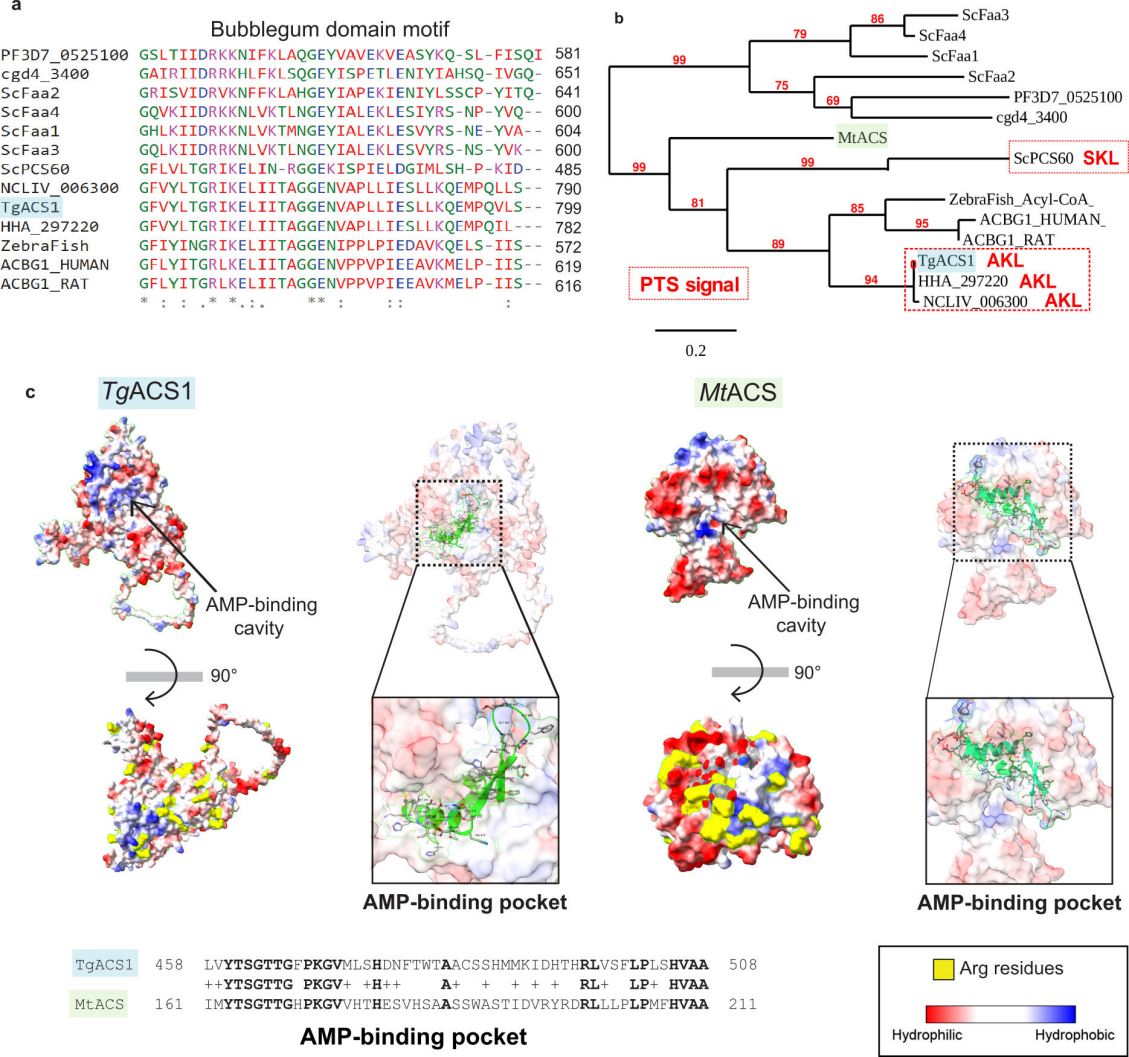
TgACS1 is a protein containing a bubble gum domain and PTS1 signal sequence which is not important for correct intracellular localization. (**a**) Phylogenetic analyses and Blastp analyses reveal the presence of a conserved bubble gum domain in this protein with high sequence similarity with closest ACS1 homologs from other organisms: *Plasmodium falciparum* (PF3D7_0525100), *Cryptosporidium parvum* (cgd4_3400), *Saccharomyces cerevisiae* (ScFaa2, ScFaa4, ScFaa1, ScFaa3, ScPCS60), *Neospora caninum* (NCLIV_006300), *Toxoplasma gondii* (TgACS1), *Hammondia hammondi* (HHA_297220), zebra fish, human (ACSBG1HUMAN), and rat (ACSBG1_RAT). The red numbers represent the bootstrap values. (**b**) A cladogram of homolog ACS proteins from other proteins shows to contain a peroxisomal targeting sequence (PTS1) tripeptide sequence (in red) is present at the C-terminal end of TgACS1 and ScPCS60, a known peroxisomal ACS. (**c**) Comparison of the three-dimensional structures of the predicted model for TgACS1 (Alphafold2; left schemes) and the 3D representation of the crystal structure from *Mycobacterium tuberculosis* ACS (MtACS, right schemes): the AMP-binding pocket is conserved between the two (carbon alpha representation) with a sequence identity of 51% (sequence alignment). Surface representations show the conservation of the hydrophobic area from the putative AMP-binding pocket in TgACS1 (blue areas) and similar arrangement of the arginine residues forming a surface cluster (yellow patches).

To identify putative functional domains in *Tg*ACS1 protein sequence, Smart Protein program (www.smart.embl-heidelberg.de) and Phyre 2 were used to compare *Tg*ACS1 with known related protein and ACSs domains. The presence of a conserved “bubble gum” domain was confirmed in *Tg*ACS1, supporting the phylogenetic analyses described earlier (Fig. 2a), and suggesting *Tg*ACS1 has a function potentially similar to an ACS with function(s) related to β-oxidation. We also identified a highly conserved PTS1 present in the C-terminal end of the protein (tripeptide, Alanine-Lysine-Leucine, AKL). The closest homologs of *Tg*ACS1 in alveolates/Apicomplexa such as in *Hammondia* (HHA_297220) and *Neospora* (NCLIV_006300) but also known peroxisomal ACSBG in *Saccharomyces cerevisiae* (ScPCS60) also contain a similar conserved PTS (Fig. 2b). However, the existence of peroxisome *per se* in *T. gondii* has long been questioned and remains a debated topic as (i) the typical organelle’s presence remains elusive or not fully defined, and (ii) there is no clear experimental evidence for biochemical activities attributed to such predicted organelle compartment(s). Interestingly the PTS found in *Tg*ACS1 is similar to the one identified in other *Toxoplasma* proteins and is able to rescue peroxisomal import assay in yeast mutant (38). Using Alphafold and a 3D crystal structure of a bubble gum domain-containing ACS found in *Mycobacterium tuberculosis* (47), which shares 51% of sequence identity with *Tg*ACS1, we thus generated a 3D model prediction (Fig. 2c). These protein structures seem to have similarly placed hydrophobic regions, suggested to be the AMP-binding cavity, and similar placement of arginine residues, which could be the site for putative membrane interaction of ACS [Fig. 2c; (47)].

### *Tg*ACS1 is critical for intracellular development in low nutrient environment

**Fig 3:**
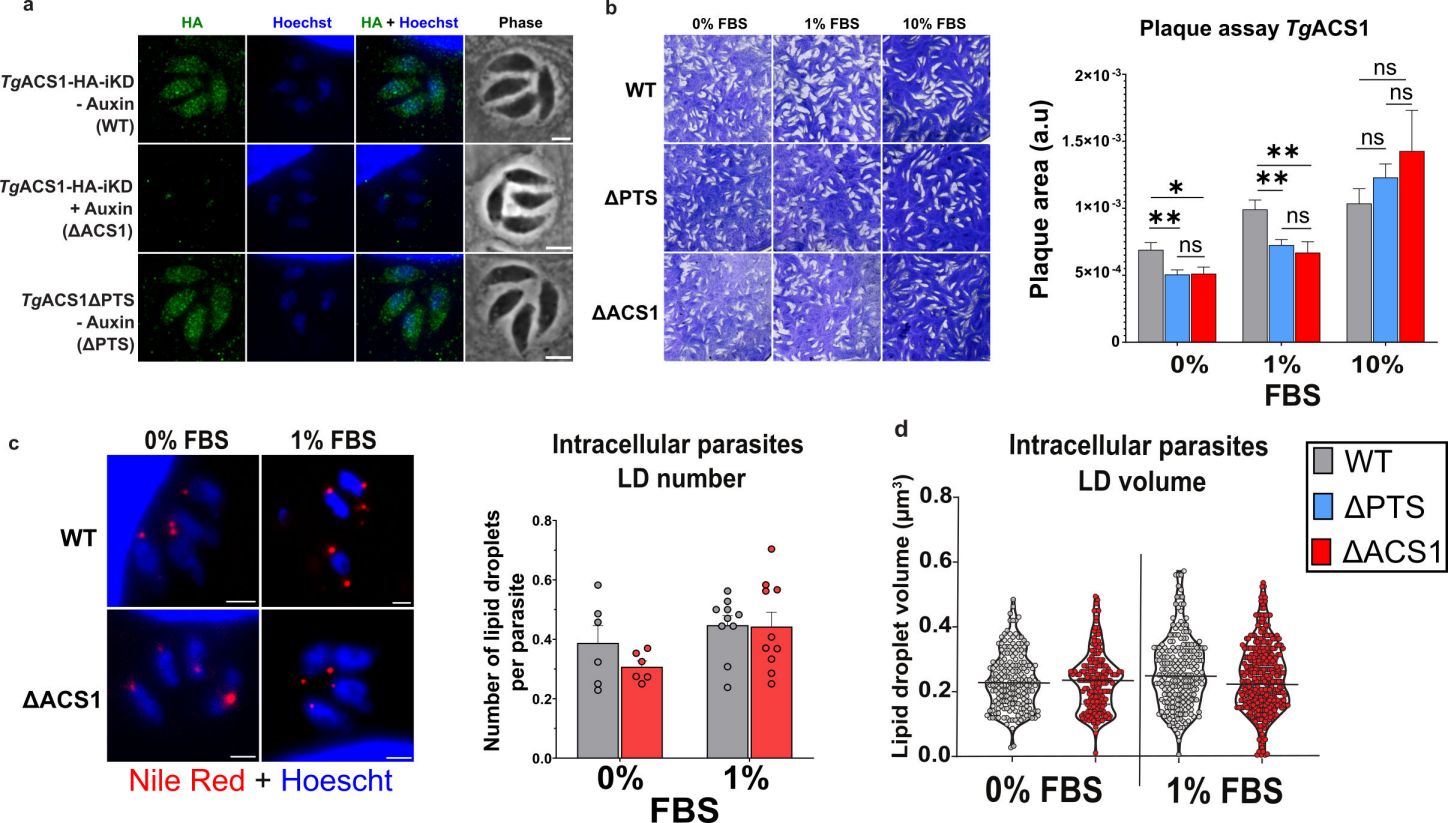
*Tg*ACS1 is important for intracellular propagation in low lipid environments. (**a**) Immunofluorescence assays of intracellular parasites reveal *Tg*ACS1 cytosolic punctate localization (in green) in Wild Type (WT) parasite (*Tg*ACS1-iKD) and in parasites lacking the predicted PTS1 (*Tg*ACS1ΔPTS). Disruption of *Tg*ACS1 is confirmed by loss of the signal in the presence of auxin (100 µM) after 24 h. (**b**) Parasite growth and survival capacities determined by plaque assay (left; i.e., measure of white lysis plaques made by parasites lysing human host cells, shown in purple) in the presence (WT) or auxin-induced absence of *Tg*ACS1 (ΔACS1), and in ACS1ΔPTS. Statistical analyses (right) reveal inhibition of parasite growth when *Tg*ACS1 is degraded (red/ΔACS1) or in the absence of the PTS signal (blue/ΔPTS) at low lipid environment: 0% and 1% FBS but not significant at 10% FBS, compared to the WT (gray/WT). (**c**) Analysis of LD content by measuring fluorescent labeling of neutral lipids revealed by Nile red (left). Statistical analysis (right) of intracellular parasites (*n* = 300) indicating the number of LD with (gray/−auxin) and without (red/+auxin) *Tg*ACS1 per parasite (*n* = 200). (**d**) LD volume analyses of intracellular parasites (*n* = 300) with (gray/−auxin) and without (red/+auxin) *Tg*ACS1. Scale bars = 2 µm. Error bars indicate standard deviation. Experiments were conducted in triplicate, unpaired *t*-test *P*-values are indicated only when statistically significant.

To determine the localization and role of *Tg*ACS1, we generated an auxin-inducible degron (AID)-based inducible knockdown mutant of *Tg*ACS1 with a C-terminal Hemaglutinin (HA) tag. The PTS was added after the HA tag, thus avoiding a potential inactivation of the PTS due to the proximity with the tag [(48); Fig. S2a]. The confirmation of the molecular constructs and its correct insertion at the endogenous locus in the parasite genome was confirmed by PCR (Fig. S2b). A similar mutant line lacking the predicted PTS1 sequence {C-terminal AKL, consensus of the tripeptide is [(S/A/C)(K/R/H)(L/M)]} was also generated, which we called *Tg*ACS1ΔPTS (Fig. S2a). Immunofluorescence microscopy assay (IFA) of *Tg*ACS1 and *Tg*ACS1ΔPTS revealed that both mutant line signals adopted a punctate localization spread throughout the cytosol (Fig. 3a), and the addition of auxin leads to the loss of the signal. The identical signal of *Tg*ACS1 and *Tg*ACS1ΔPTS suggested that the PTS was not important for correct intracellular localization of this protein. We then confirmed the mutant strain by Western blot analysis confirming the expected protein size of the HA-tagged *Tg*ACS1-iKD at ~100 kDa (Fig. S2c). The downregulation of *Tg*ACS1 by the addition of 100 µM auxin was confirmed by Western blot and IFA [1% fetal bovine serum (FBS), Fig. S2c and Fig. 3a], with a complete degradation of the protein within 1 h of auxin treatment (Fig. S2c). To determine if the depletion of this protein influenced parasite intracellular growth, a replication assay was performed and showed a slight yet non-significant increase in the number of small vacuoles (one to four parasites per vacuole) and a decrease in that of larger vacuoles (10 parasites or more per vacuole) upon *Tg*ACS1 degradation (Fig. S2d).

Since the host nutrient environment affects the development and metabolism of the parasite, as shown in our previous work (7, 18), the parasites were grown in different concentrations of FBS to simulate different physiological nutrient levels. We performed plaque assays of *Tg*ACS1 and *Tg*ACS1ΔPTS in different host nutritional conditions, i.e., at 0%, 1%, or 10% FBS in Dulbecco’s Modified Eagle’s Medium (DMEM) as previously described (7). There was a significant difference in plaque size, and hence intracellular development of the parasite, specifically under low (1% FBS) and very low (0% FBS) nutrient/lipid conditions. Indeed, at 0% and 1% FBS, when disrupting *Tg*ACS1 (red/+auxin/ΔACS1), plaques lysis area was significantly smaller than those of the WT (gray/−auxin, Fig. 3b). Interestingly, when only the PTS1 of *Tg*ACS1 was removed (blue/−auxin/ΔPTS), same plaques lysis area as ΔACS1 was observed (Fig. 3b), suggesting that the PTS is important for the intracellular function of ACS1, which becomes important for the parasite in low nutrient environment. At 10% FBS, parasites grew normally even without the protein (Fig. 3b), indicating that *Tg*ACS1 is not important in high nutrient conditions.

Since *Tg*ACS1-iKD grows differently in response to nutrient availability and belongs to the ACSBG subfamily, it is possible that the protein has a role in the flux or storage of FA. LDs are the main source for storage lipids (diacylglycerol, DAG, and triacylglycerol, TAG) and FA in the parasite. In other eukaryotic models, LD can provide FA to peroxisomes, where they can be used for energy production through β-oxidation (49). Hence, we decided to analyze how LDs were affected in the absence of *Tg*ACS1 in intracellular parasites. Here, we used Nile red to visualize LDs in various nutrient conditions (0%–1% FBS). The results showed no significant difference in the number of LD per parasite with or without *Tg*ACS1 (Fig. 3c), and no change in LD volume (Fig. 3d), suggesting that *Tg*ACS1 might not be important for LD formation or mobilization during intracellular life stages.

### The disruption of *Tg*ACS1 impacts the free fatty acid content of the parasite under low nutrient conditions

Since there was no difference in LD number between parasites with or without *Tg*ACS1, we investigated whether there was any change in the parasite’s lipidomic profile. To that end, we conducted lipidomic analyses by gas chromatography coupled to mass spectrometry (GC-MS) and separation of neutral lipid classes (TAG, DAG), FFAs, and all phospholipids (PLs) using high-performance thin layer chromatography (HPTLC) on the *Tg*ACS1-iKD intracellular parasites.

**Fig 4:**
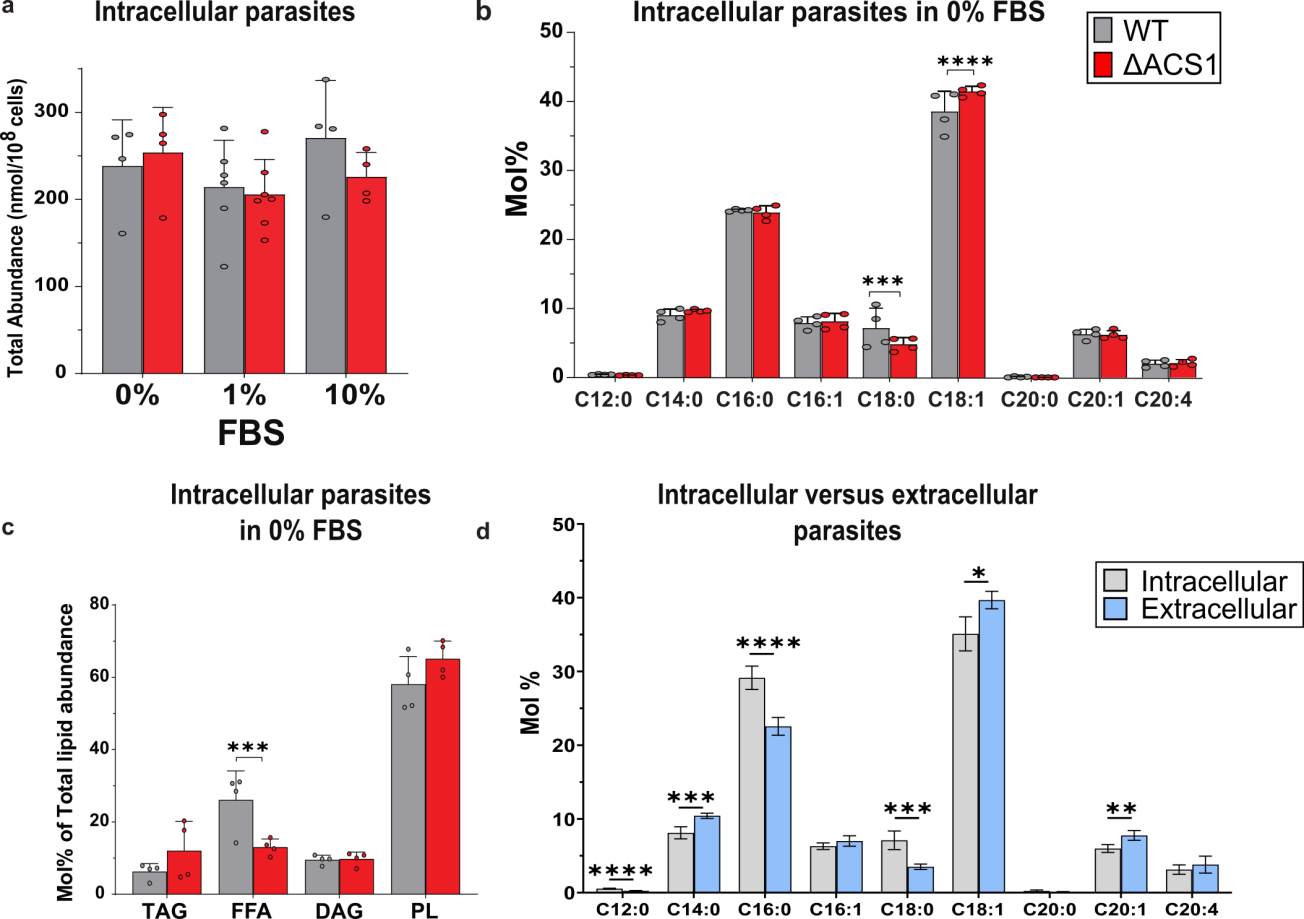
The disruption of *Tg*ACS1 impacts the free fatty acid content of the parasite under low nutrient conditions. (**a**) Total lipid abundance (nanomole per cell number) of intracellular in low, normal, and high FBS (0%/1%/10%) with (gray/−auxin) and without (red/+auxin) *Tg*ACS1. (**b**) FA profile (mol%) of intracellular parasite in 0% FBS with (gray) and without (red) *Tg*ACS1. (**c**) Neutral lipid mol% of total lipid abundance in intracellular parasites in 0% FBS with (gray/−auxin) and without (red/+auxin) *Tg*ACS1, namely TAG, FFA, DAG, and PL. (**d**) FA profile (mol%) of intracellular (gray) and extracellular (blue) WT parasites in 1% FBS. Error bars indicate standard deviation, experiments were conducted in triplicates, unpaired *t*-test *P*-values are indicated only when statistically significant.

Firstly, we examined the lipidomic profiles of intracellular parasites at 0%, 1%, or 10% FBS (Fig. 4a). There was no change in total FA abundance when the protein was depleted, which was expected since we saw no changes in LD formation in the Nile red analyses (Fig. 3c and d). To further investigate the nutrient-dependent role of *Tg*ACS1, we quantified the phospholipid and neutral lipid content of intracellular parasites in different nutritional conditions (namely 0%–1%–10% FBS, Fig. 4b and c; Fig. S3a through g).

Under low nutrient content (0% FBS), there was a significant increase of the relative abundance (mol%) of oleic acid, C18:1, concomitant to a significant decrease of C18:0 in the absence of *Tg*ACS1 (Fig. 4b). To further determine the precise function of *Tg*ACS1, the total lipid fractions were separated by HPTLC and the composition and content of total PLs, TAG (i.e., LD component), DAG, and FFAs were quantified by GC-MS. This lipidomic analyses revealed that in the absence of *Tg*ACS1, there was a slight yet no significant increase in TAG levels, concomitant to a significant decrease in FFA levels, only in low nutrient content (0% FBS) (Fig. 4c). This suggests that *Tg*ACS1 may allow the marginal activation of FA from the mobilization of TAG storages in parasite LD, in intracellular parasites under low nutrient content .

At 1% FBS, intracellular parasites lacking *Tg*ACS1 displayed no change in their total lipid fatty acyl profile. Additionally, there was no noticeable change in neutral lipid content when the protein was knocked down under the same conditions (Fig. S3a and b). Altogether, these results show no evidence that *Tg*ACS1 is involved in LD mobilization in intracellular parasites. Yet, the growth defect observed at 0% and 1% FBS prompted us to check whether there were specific conditions for which *Tg*ACS1 might be more important for the parasite. One such example is the difference in conditions for the parasite when it’s intracellular versus extracellular. Indeed, nutrient availability and physiological conditions are drastically different in these conditions. To go further, we performed lipidomic analysis on intracellular and extracellular parasites, and determined their respective lipid profile under normal nutrient condition (1% FBS). Very interestingly, the FA composition of extracellular parasites significantly differed from that of intracellular parasites. There was significantly higher relative abundance (mol%) of C14:0, C18:1 and C20:1, whereas there were significantly less C12:0, C16:0, and C18:0 in extracellular parasites compared to intracellular ones (Fig. 4d). Based on these differences, it is possible that *Tg*ACS1 could be rather involved in the parasite’s extracellular life stage when nutrients are scarce.

### *Tg*ACS1 is relocated via its PTS1 in extracellular stage where it is essential for parasite motility

**Fig 5:**
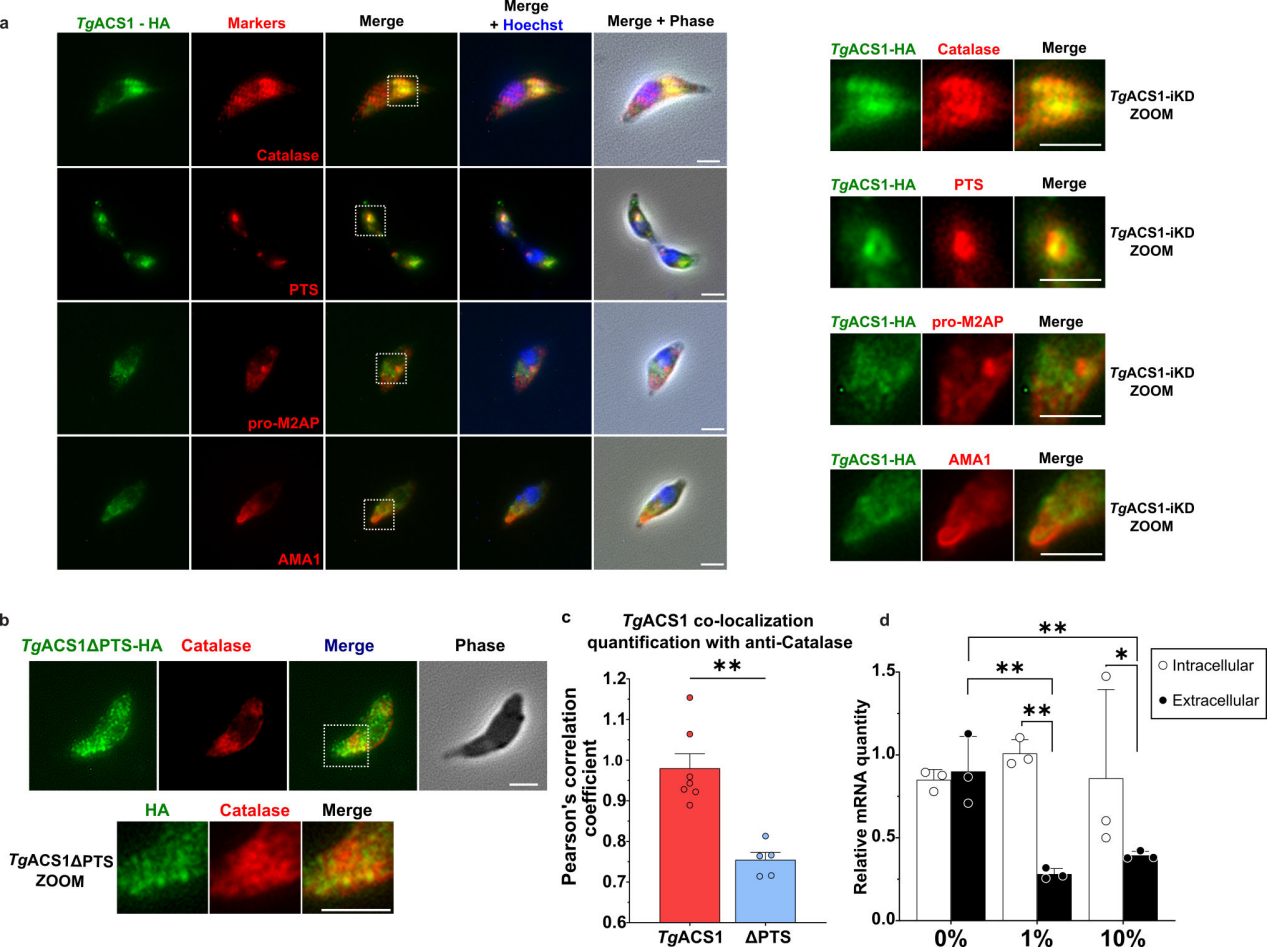
The predicted PTS1 is needed for correct localization of TgACS1 in extracellular parasites. (**a**) IFAs of extracellular parasites at 1% FBS show co-localization/close proximity of TgACS1 (in green) with two peroxisomal-like markers (anti-TgCatalase and GFP-ePTS, in red), but did not show overlapping signals with the early and late endosome-like compartments marker (anti-proM2AP, in red) or micronemes marker (anti-AMA1, in red); see enlarged areas (yellow squares) on the right-hand side panels. (**b**) IFAs of extracellular parasites at 1% FBS show a scattered cytosolic signal for TgACS1∆PTS (in green), not co-localizing with TgCatalase (in red). (c) Quantification of co-localization levels between (**I**) TgCatalase and TgACS1-mAID-HA (in red/TgACS1), and between TgACS1ΔPTS-mAID-HA and catalase (in blue/ΔPTS), using Person’s correlation coefficient. (**d**) Relative mRNA expression of TgACS1 (compared to TgGADPH, a housekeeping gene for which value is 1.0) in intracellular (white) and extracellular (black) parasites in different FBS conditions (0%–1%–10%). Scale bars = 2 µm. Error bars indicate standard deviation (SD). Experiments were conducted in triplicate, unpaired *t*-test *P*-values are indicated only when statistically significant.

Although parasites stay extracellular for a short period of time, their fitness and motility during this stage are highly important as it facilitates the search for a new host allowing for parasite propagation. This step involves energy-demanding pathways (such as motility gliding) in a nutrient-deprived environment. Based on the intracellular phenotype in the absence of *Tg*ACS1, we sought to determine whether *Tg*ACS1 had a role in extracellular stage of the parasite. We first assessed the localization of the protein by IFA. Very interestingly, IFAs revealed that *Tg*ACS1 adopts a different localization in extracellular conditions than in intracellular tachyzoite stage. Once extracellular, *Tg*ACS1 is relocated from its scattered punctate cytosolic intracellular localization to larger compacted vesicular formations remobilized toward the anterior part of the parasite, most often located between the nucleus and the apicoplast, in the ER-Golgi-like area, and reminiscent of the previously reported *Tg*Catalase localizations (Fig. 5a). Only a handful of putative peroxisomal *Toxoplasma* proteins have been experimentally assessed so far, with nuanced results regarding their localization: *Tg*Catalase was described to be cytosolic but looked like it could rather be close to a Golgi-like position, and biochemical fractionation revealed a dual cytosolic and organellar localization in *T. gondii* (34). Other studies pointed again at an ER/Golgi-like localization of *Tg*Catalase, and bioinformatic analyses concluded that *Tg*Catalase is most likely present in peroxisome-like structures in *T. gondii* (35, 38). Similarly, other putative peroxisomal proteins, *Tg*SCP2 and *Tg*PEX5, revealed a dual cytosolic/punctate, and specifically more compact punctate localizations in the anterior end of the parasite, respectively (17, 35, 38). To further determine the specific localization of *Tg*ACS1, we performed IFA co-localizations with the anti-*Tg*Catalase antibody on extracellular parasites. This confirmed and determined that catalase adopts a similar cytosolic-apical compact structure to *Tg*ACS1 (Fig. 5a; Fig. S4a), confirming previous results (34). Previous studies also showed that *Toxoplasma* PTS is sufficient to send *Toxoplasma* proteins to peroxisomal-like punctate (co-localizing with the catalase and PEX5). The PTS is also sufficient to import *Toxoplasma* catalase to eukaryotic peroxisomes in heterologous localization approaches (35, 38). Therefore, to support the hypothesis that *Tg*ACS1 localizes to peroxisome-like structure/area, a GFP tagged with an enhanced PTS (GFP-ePTS) was transiently expressed in *Tg*ACS1-iKD. In extracellular parasites, IFA revealed a punctate localization of GFP-ePTS overlapping almost perfectly with *Tg*ACS1, further strengthening the hypothesis for the localization of *Tg*ACS1 in a peroxisomal-like compartment, pending confirmation of the existence of such compartment in *Toxoplasma* (Fig. 5a). More detailed IFA co-localization analysis showed no co-localization with apical endosome-like compartments (anti-proM2AP) or apical organelles of the parasites, such as micronemes (anti-AMA1, i.e. Apical Major Antigen1), (Fig. 5a). To confirm that the delocalization of the protein observed in such condition was not a delocalization of peroxisome-like *per se* in extracellular stages, we conducted IFA in intracellular parasites showing a similar localization of *Tg*Catalase as in extracellular parasite (Fig. S4b). Very importantly, when the PTS is removed as (*Tg*ACS1ΔPTS), the protein loses its compact anterior cytosolic localization to be re-distributed to a spread cytosolic/punctate as in that of *Tg*ACS1-iKD in intracellular parasites (Fig. 5b). This points at the direct role of the PTS for *Tg*ACS1 correct localization. The level of co-localization of *Tg*ACS1-iKD with *Tg*Catalase was further quantified using Pearson’s correlation coefficient, which gives the correlation between the intensity of each channel in each pixel. Pearson’s correlation coefficient varies from 0, reflecting no correlation, to 1, reflecting perfect positive correlation. Pearson’s correlation coefficient between *Tg*ACS1-iKD and *Tg*Catalase in extracellular parasite was measured around 1, reflecting an almost perfect overlapping of both channels (Fig. 5c). However, the coefficient between *Tg*ACS1ΔPTS and *Tg*Catalase was significantly reduced (around 0.75; Fig. 5c), suggesting that both signals could localize at certain spots/volume but not as perfectly as that of *Tg*ACS1 bearing a PTS, and *Tg*Catalase. This quantification thus strongly suggests that (i) *Tg*ACS1 re-localizes to a similar structure at *Tg*Catalase in the anterior end of the parasite, and which most likely defines as a peroxisomal-like zone or structure, whether it is membrane bound or not, and (ii) the presence of the PTS is necessary for the protein to be sent to this compact anterior structure/area. This re-localization of *Tg*ACS1 in extracellular parasites also further highlights its importance in low nutrient conditions during extracellular stages.

The AID construction allowed us to maintain *Tg*ACS1 under the control of its endogenous promoter. This allowed us to check if the nutritional status of the host or the environment had an impact on the gene expression profile of *Tg*ACS1, which could correlate to the observed phenotypes. To that end, we assessed the expression levels of *Tg*ACS1 in intracellular vs extracellular tachyzoite under different nutritional conditions. We found that in extracellular parasites, *Tg*ACS1 mRNA levels were significantly higher at 0% compared to 1% and 10% FBS. In intracellular parasites, the levels of expression of *Tg*ACS1 stayed stable under different nutrient conditions (Fig. 5d). Taken together, this confirms our observations in the role and importance of *Tg*ACS1 during the extracellular stage of the parasite, under low nutrient conditions.

The extracellular stage is critical for the parasite, as it constitutes a very short window of time during which the parasite needs to quickly find a new host cell to invade, through energy-dependent gliding motility, and under limited resources. Very little is known of extracellular parasite metabolism except that they can be independent from external carbon sources for motility and ATP generation (39). Instead, the parasite can use its own internal storage like GABA, made from glutamine when intracellular, to maintain motility in extracellular stages by fueling the TCA cycles, generating the reducing power for ATP synthesis (40). They can also partly use internal glucose within the first hour of their extracellular life to maintain ATP homeostasis and gliding motility (39).

**Fig 6:**
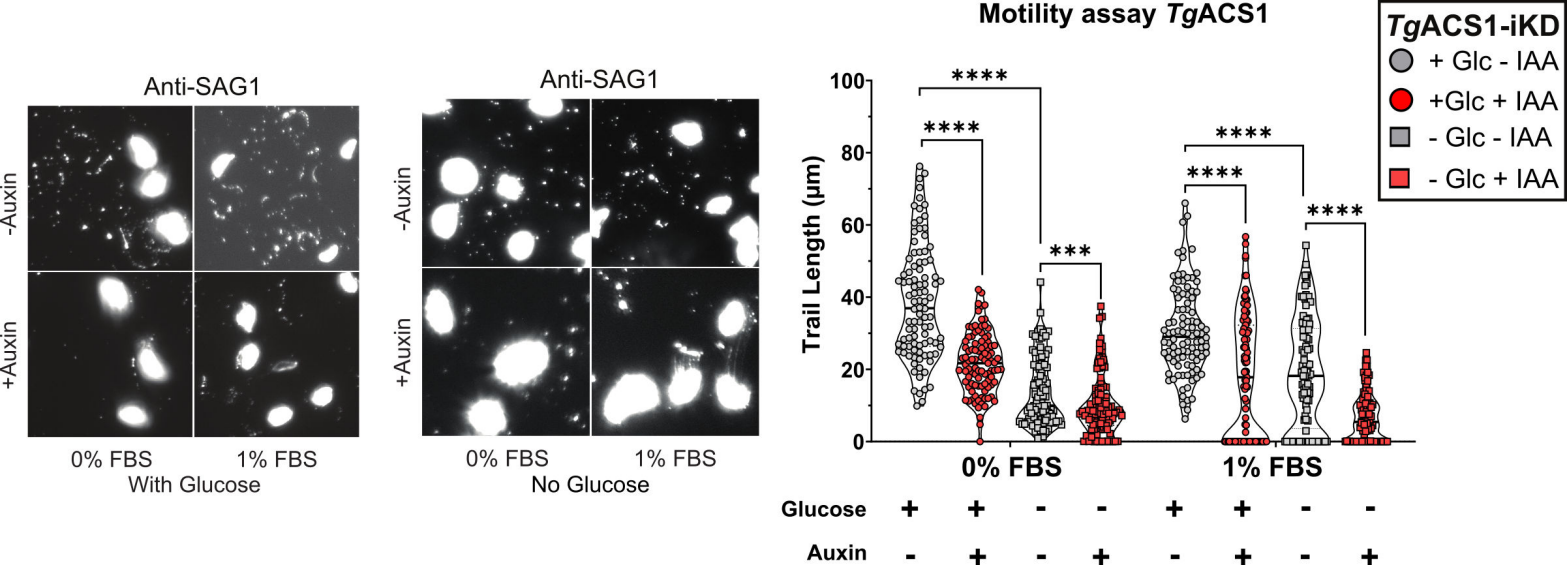
TgACS1 is crucial for extracellular parasite motility. Quantification of parasite motility capacities (gliding motility assay) by measuring parasite gliding trails revealed by probing anti-SAG-1 via IFA (lefts panels), at 0% and 1% FBS, with and without glucose (left), and its quantification and statistical analysis (right) representing the length of gliding trails per extracellular parasite (*n* = 50) with (in gray/−IAA) and without (in red/+IAA) TgACS1. Error bars indicate standard deviation (SD). Experiments were conducted in triplicate, unpaired *t*-test *P*-values are indicated only when statistically significant.

Based on its predicted function for β-oxidation and its localization in peroxisome-like structure in extracellular parasites, we thus investigated if *Tg*ACS1 could be involved in energy production in the parasite. We analyzed the viability of energy-producing state of *Tg*ACS1-ikD parasites under “stressful” conditions that may potentially induce β-oxidation, i.e., without nutrients and outside the host. We performed parasite gliding (motility) assays of extracellular parasites in different nutrient conditions (0%–1% FBS with and without glucose), and we measured the motility trails lengths formed by the release of surface antigen 1 (SAG1) during parasite gliding motility in its extracellular stages. It revealed that in the absence of *Tg*ACS1, parasite motility was significantly decreased in terms of average trail length (µm) in all conditions (Fig. 6). When measuring the motility rate (nominal data), we noticed that only in low nutrient conditions [0% FBS and phosphate-buffered saline (PBS)] does motility diminish for extracellular parasites (Fig. S4c). Interestingly, in the absence of glucose (which is the primary source for ATP production, i.e., glycolysis), it was noted that the overall average slime trail length was decreased when compared to condition with glucose by twofold, and this was more pronounced when *Tg*ACS1 was absent, in both 0% and 1% conditions (Fig. 6). Together, this confirms previous results on the role of glucose for parasite motility but, most importantly, shows that *Tg*ACS1 is actually involved in an energy production process in extracellular parasites.

### *Tg*ACS1 mobilizes LD for energy production in extracellular parasite

**Fig 7:**
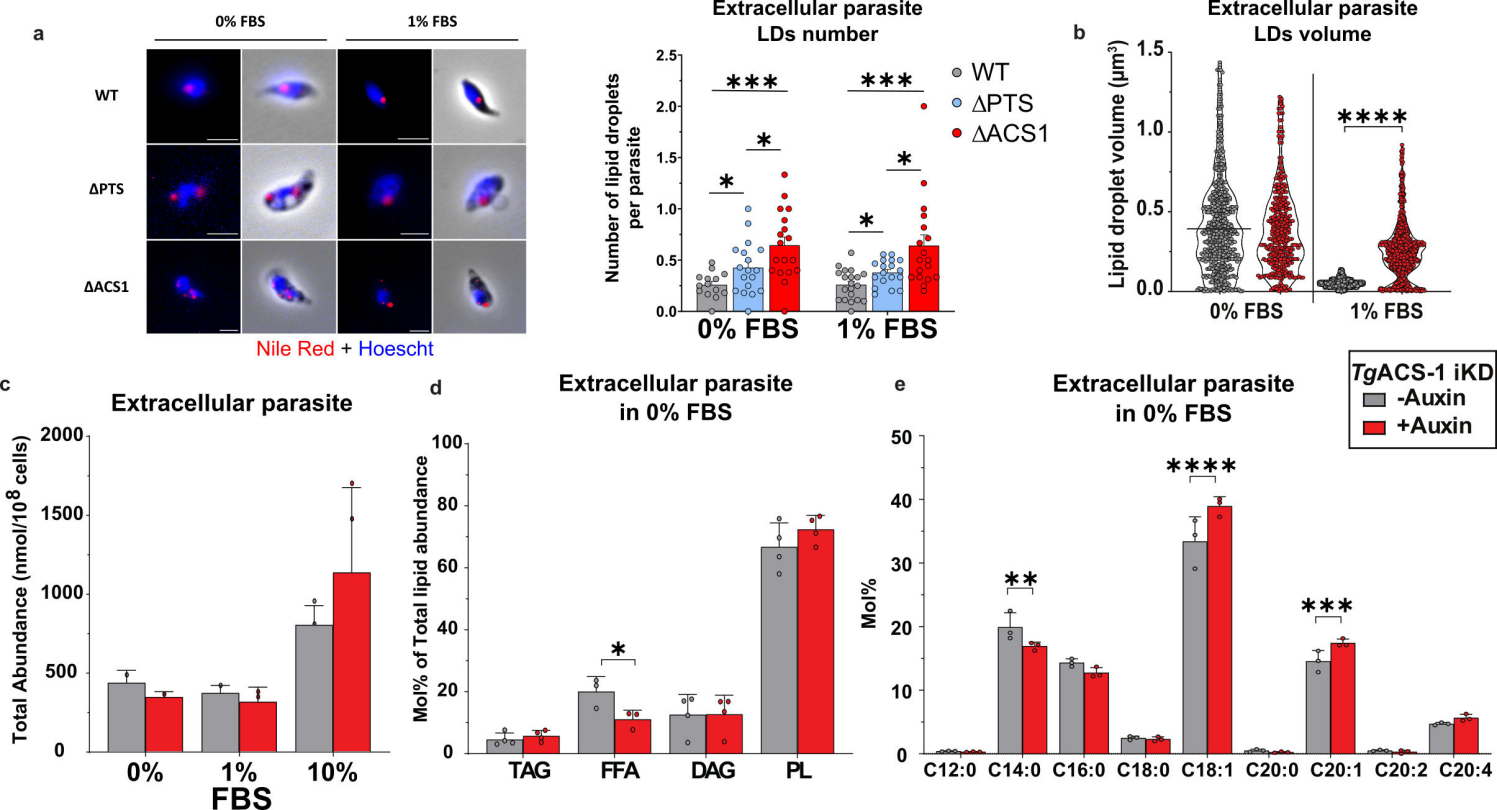
*Tg*ACS1 is important for extracellular parasite LD utilization for energy production. (**a**) Fluorescence microscopy using Nile red staining LD (left), with an accompanying graph (right) representing LD number per extracellular parasite (*n* = 200) at 0% and 1% FBS, with (gray/WT/−auxin) and without (red/ΔACS1/+auxin) *Tg*ACS1, and without the PTS (blue/ΔPTS/−auxin). (**b**) Further Nile red analyses with (gray/−auxin) and without (red/+auxin) *Tg*ACS1 representing LD volume at 0% and 1% FBS in extracellular parasites (*n* = 300). (**c**) Total lipid abundance of extracellular parasite in 0%/1%/10% FBS. (**d**) Neutral lipid mol% of total lipid abundance in extracellular parasites in 0% FBS with (gray/−auxin) and without (red/+auxin) *Tg*ACS1, namely TAG, FFA, DAG, and PL. (**e**) FA profile (mol%) of extracellular parasite in 0% FBS with (gray/−auxin) and without (red/+auxin) *Tg*ACS1. Scale bars = 2 µm. Error bars indicate standard deviation (SD), experiments were conducted in triplicate, and unpaired *t*-test *P*-values are only indicated when statistically significant.

The change in localization of the *Tg*ACS1 protein in extracellular parasites, its predicted function as a bubble gum ACS, and the negative impact of *Tg*ACS1 disruption on high-energy demanding process, such as gliding, further prompted us to assess the impact of *Tg*ACS1 disruption on the energy storage of the cell containing lipids usually used for β-oxidation, LD, but this time in extracellular parasites. Interestingly, when the PTS is removed, the number of LD per extracellular parasite was significantly higher than in the WT under low nutrient conditions, 0% and 1% FBS conditions (Fig. 7a). This accumulation of LD was even more pronounced when *Tg*ACS1 was disrupted (Fig. 7a), together with an increase in LD volume at 1% FBS (Fig. 7b). These results highlight two important points: (i) that the absence of re-localization of *Tg*ACS1 due to the absence of its PTS impairs LD biogenesis in extracellular parasite and low nutrient content, and (ii) that the complete suppression of *Tg*ACS1 results in a significant accumulation of LDs. Together, this indicates that in the absence of *Tg*ACS1, LDs are not consumed/utilized as usual, thus increasing in size and volume, which likely points to a role of the protein in the utilization of LD content potentially for energy production for extracellular parasites motility.

Due to the changes observed in LD number and volume, we investigated the lipidomic profile of extracellular parasites in *Tg*ACS1. By doing so, we also revealed the lipidome of the *T. gondii* tachyzoite extracellular stages. Surprisingly, there was no change in the total FA abundance of extracellular parasites at 0%–1%–10% FBS (Fig. 7c) in the absence of *Tg*ACS1. Furthermore, just as in intracellular parasites (Fig. 4c), we noticed a significant decrease in FFA levels in extracellular parasites in 0% FBS (Fig. 7d; Fig. S5e and f), a slight but not significant decrease at 1% FBS (Fig. S5b and g), but no differences at 10% FBS (Fig. S5c and d). These data collectively indicate that *Tg*ACS1 plays an important role in low nutrient conditions, specifically in the mobilization of acyl chains from TAG/lipid storage and for the formation of FFAs.

We quantified the FA species abundance of extracellular parasites in different nutrient conditions. In extracellular parasites at 0% FBS, *Tg*ACS1 depletion shows a significant increase of C14:0 and a decrease of C18:1 and C20:1 (Fig. 7e). At 1% FBS under depletion of the *Tg*ACS1, there is a similar trend, wherein there is an increase in C18:0 and C16:0 and a decrease in C18:1, suggesting *Tg*ACS1 has a substrate specificity to these FA chains (Fig. S5a).

Overall, these results suggest that *Tg*ACS1 plays a more important role in the activation of FFA in extracellular parasites under low nutrient conditions, and that re-localization of the protein is needed for the biogenesis of LDs that the parasite will use in such conditions. Moreover, this protein seems to have a substrate specificity for longer, unsaturated FA species that are normally derived from externally acquired lipids/FA.

### *Tg*ACS1 is capable of rescuing yeast strain defective of the peroxisomal bubble gum acyl-CoA synthase function linked to β-oxidation but cannot complement any other ACS functions

To further confirm the acyl-CoA synthase activity of *Tg*ACS1 and determine its precise function, we performed different heterologous complementation of *Saccharomyces cerevisiae* yeast strains defective for different ACS classes and functions. In yeast, it is known that acyl-CoAs can be made, either (i) directly by the endogenous FAS pathway or (ii) by the activation of exogenous fatty acyl chains by ACSs.

**Fig 8:**
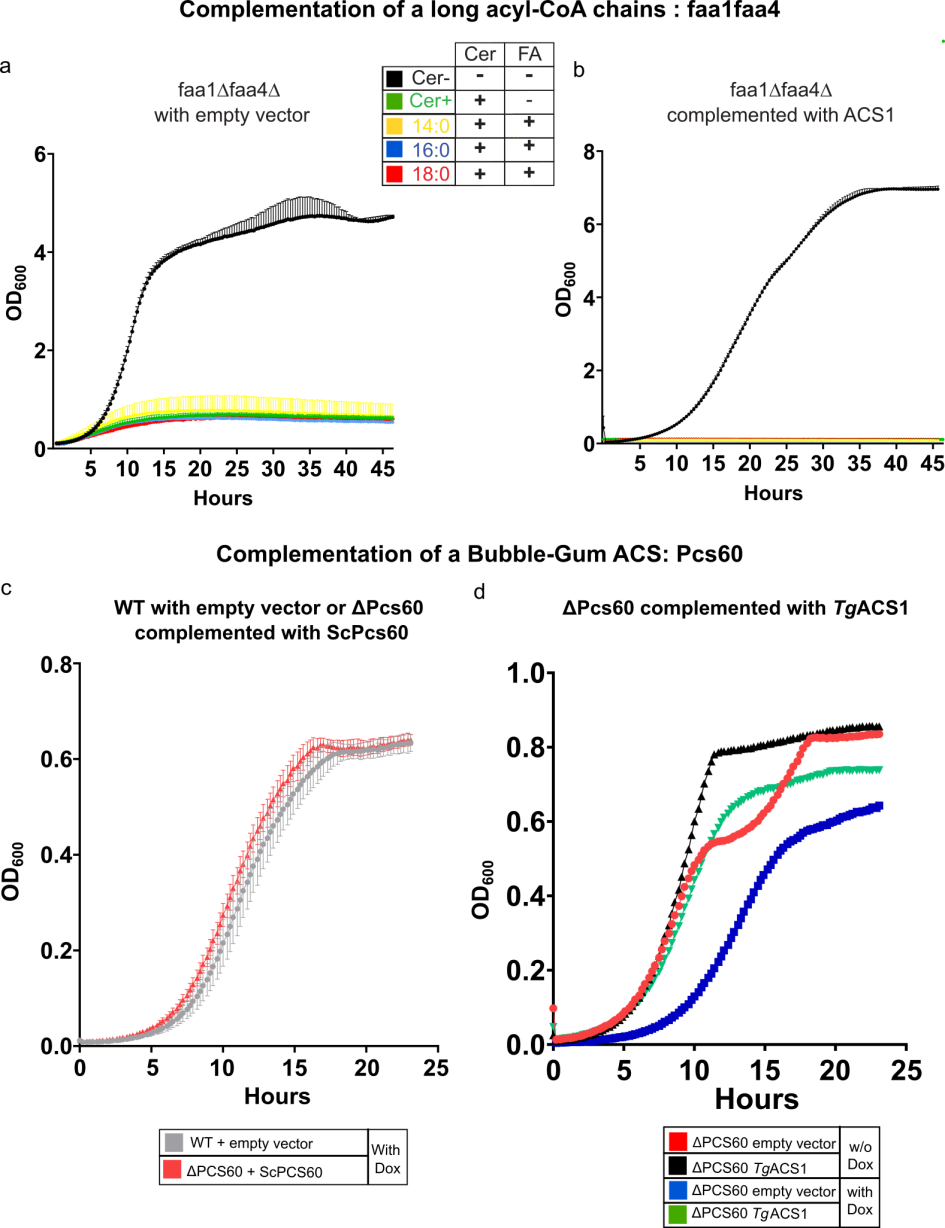
Growth complementation shows that *Tg*ACS1 is an ACS that can rescue the activity of a yeast mutant deficient of peroxisomal acyl-CoA activity. (**a**) Growth complementation of a yeast mutant strain deficient of long acyl-CoA chains activity (faa1Δfaa4Δ) complemented with an empty vector. This yeast strains were grown in a glucose (2%) and Tween 20 (0.2%, to induce β-oxidation) -containing medium(Cer-, black, positive control to allow for FA synthesis), with the addition of 22.5 µM cerulenin only (Cer+, green, negative control), or additionally in the presence of 100 µM of FFAs C14:0 (yellow), C16:0 (blue), or C18:0 (red), respectively. (**b**) Growth curves of a *Tg*ACS1 complemented faa1∆faa4∆ double yeast mutant in a glucose (2%) and Tween 20 (0.2%) media supplemented with 22.5 µM cerulenin and 100 µM of FA as before (C14:0/yellow, C16:0/Blue, C18:0/red). Medium with 22.5 µM cerulenin but FFA was used as a negative control (black). (**c**) Growth complementation of a the WT yeast strain BY4741 with an empty vector (in gray), and the yeast mutant strain deficient of peroxisomal acyl-CoA activity (ΔPCS60) complemented with the yeast *Sc*Pcs60 (in red), in the presence of doxorubicin (12 µg/mL, 30°C for 24 h). (**d**) Growth complementation of a yeast mutant strain deficient of peroxisomal acyl-CoA activity (ΔPCS60) expressing *Tg*ACS1 (in black/−doxorubicin, in blue/+doxorubicin) or not (empty vector, in red/−doxorubicin, in green/+doxorubicin).

Johnson et al. 50) have shown that the two major yeast ACSs, Faa1 and Faa4, are involved in the activation of long acyl-CoA chains (i.e., part of the Very Long Chain or VLC-ACS subfamily). Indeed, when FA synthesis is inactivated by the addition of cerulenin, a FAS inhibitor, the double mutant strain *faa1*∆*faa4*∆ can no longer grow, and this growth defect is not rescued by addition of exogenous FAs. To test whether *Tg*ACS1 could act as a long FA activator (i.e., acting as an ACSL, similar to FAA1 and FAA4 in yeasts), we transformed the *faa1*∆*faa4*∆ double mutant yeast strain (*faa1*∆*faa4*∆) with a pRS high copy (*ura3*) plasmid expressing *Tg*ACS1. A double mutant strain was also transformed with an empty vector (no *Tg*ACS1) used as a negative control. All experiments were performed in synthetic Yeast Nitrogen Base (YNB) media without uracil, supplemented with glucose (2%) and Tween 20 (0.2%, to induce β-oxidation). The *faa1*∆*faa4*∆ strain not complemented with *Tg*ACS1 displayed the expected phenotype: it was able to grow normally when the FAS pathway is active (in black, Fig. 8a). The inhibition of FAS by the addition of cerulenin led to a significant decrease of the growth rate of this strain (in green, Fig. 8a). As previously described, under cerulenin treatment, there was no rescued phenotype by addition of any FA species (in yellow, blue, and red, Fig. 8a). Importantly, the same results were obtained in the yeast mutant strain *faa1*∆*faa4*∆ complemented by *Tg*ACS1: the complemented strain was able to grow normally in the absence of cerulenin treatment (in black, Fig. 8b), and cerulenin treatment with or without addition of FA species leads to a decreased growth rate (Fig. 8b; Fig. S6a through c). These results suggest that *Tg*ACS1 is not able to rescue the activity of a yeast mutant strain deficient of long acyl-CoA chain activity, and thus, that *Tg*ACS1 is not an ACS that depends on exogenously acquired FFAs (28, 51).

Since no complementation was seen in the *faa1*∆*faa4*∆ strain, we investigated another possible acyl-CoA synthetase activity for *Tg*ACS1 that is more linked to its predicted function and homology. Indeed, the cladding of TgACS1 as a bubble gum ACS, its localization in a peroxisomal-like compartment, and its involvement in parasite motility when extracellular prompted us to perform heterologous complementation in a mutant lacking ACSBG function. To do so, we used a yeast strain lacking the peroxisomal-CoA synthetase, ∆PCS60, the yeast’s closest homolog of *Tg*ACS1 (Fig. 1b), belonging to bubble gum subfamily ACSBG, and furthermore allowing β-oxidation in yeast peroxisome (26, 52). To test whether *Tg*ACS1 is an ACS that can rescue the activity of a yeast mutant deficient of peroxisomal acyl-CoA activity, we used the *Saccharomyces* PCS60 deficient strain in the presence of doxorubicin (12 µg/mL), a cancer drug inducing lipid peroxidation that is lethal in the absence of PCS60 (32, 53). To determine the concentration of doxorubicin to be used for our experiments, we grew ∆Pcs60 in the presence of different concentrations (6, 12, and 24 µg/mL). We determined that from 12 µg/mL of doxorubicin, the drug significantly affected ∆Pcs60 yeast strain growth (Fig. S6d), which we used throughout the complementation assay. We first confirmed that the WT strain was able to grow normally under standard culture conditions in the presence of doxorubicin (in gray, Fig. 8c). Additionally, we complemented ∆PCS60 yeast strain with ScPcs60 as positive control, and confirmed that they were growing as expected the same way as the WT in the presence of doxorubicin (in red and blue, respectively, Fig. 8c). Interestingly, the complementation of ∆PCS60 yeast strain with ∆PCS60 lacking its PTS (∆Pcs60∆SKL) depicted a slight growth defect where it was not able to fully rescue the growth defect in the presence of doxorubicin, further pointing at the importance of the PTS for the activity of Pcs60 (Fig. S6e). We then transformed *Tg*ACS1 expressing vector (pRS426-MET25-*Tg*ACS1) into the *∆*PCS60 yeast strain similarly to what has been done for the *Magnaporthe oryzae* PCS60 homolog involved in LD mobilization and fatty acid activation for β-oxidation (32). We first confirmed that ∆PCS60 yeast strain was able to grow normally without doxorubicin (in red, Fig. 8d), and the addition of doxorubicin had a deleterious effect on the *∆*PCS60 yeast growth (in blue, Fig. 6d). We then complemented the strain with our *Tg*ACS1, and, in the absence of doxorubicin, ∆PCS60-*Tg*ACS1 grew totally normally (in black, Fig. 8c). Interestingly, complementation of the ∆PCS60 strain with *Tg*ACS1, and in the presence of doxorubicin, led to a significant and almost complete rescue of the growth phenotype observed in the absence of PCS60 and the presence of the drug (in green, Fig. 8c). Finally, when ∆PCS60 yeast strain was complemented with *Tg*ACS1 lacking its PTS (*Tg*ACS1∆PTS), there was only a partial growth similar to that of ∆Pcs60∆SKL in the presence of doxorubicin, further highlighting the importance of the PTS for the activity of the protein *Tg*ACS1 (Fig. S6f). Together with the negative results of the complementation in *faa1*∆*faa4*∆, (VLC-ACS), and the rescue of the growth phenotype of PCS60, this likely confirms that TgACS1 is a homolog to ACSBGs involved in peroxisomal β-oxidation. These results further support the hypothesis that *Tg*ACS1 is involved in FA mobilization from LD in deficient environments and extracellular parasites, for energy production probably via β-oxidation in a peroxisome-like structure/area.

## DISCUSSION

Acyl-CoA synthetases catalyze a fundamental and limiting reaction in FA metabolism: the activation of free FA by addition of CoA via thioesterification (Fig. 1a). This key reaction allows the newly activated FA, i.e., fatty acyl-CoA intermediates, to participate in trafficking, assembly into complex lipids, post-translational modification of membrane proteins, and/or the catabolism of FAs via β-oxidation (20).

Our phylogenetics analysis revealed the presence of seven likely non-redundant ACSs in *T. gondii*, pointing to the importance and complexity of lipid acquisition in this obligate intracellular parasite. Interestingly, the segregated sub-grouping of *Tg*ACS7, alongside its homologs from *Coccidia* and *Chromerida,* may indicate a specific coccidian-photosynthetic origin of *Tg*ACS7 that was potentially lost during the evolution of hemosporidians (*P. falciparum*), and may indicate particular sub-functions in these groups.

The localization of ACSs can steer the fate of acyl-CoAs by allowing ACSs to interact with different proteins involved in their direct downstream processing. For example, human ACSL1 co-immunoprecipitates with mitochondrial outer membrane proteins carnitine palmitoyl transferase 1a (CPT1a) and voltage-dependent anionic channel (28, 50, 54). No CPT1a homolog exists in Apicomplexa parasites (8). However, recent data now provide evidence for the presence of an active acyl-CoA binding protein-2 that localizes to the mitochondria of the parasite (17, 19), which is involved in FA transport within the parasite mitochondria. But whether they participate in mitochondrial membrane biogenesis and/or in the debated apicomplexan mitochondrial β-oxidation is not known. Yet, our results instead suggest that the parasite would favor a non-mitochondrial β-oxidation system fueled by *Tg*ACS1, which would likely happen in an area/structure similar to peroxisomes, yet diverge from the typical compartment found in other eukaryotes. This could thus explain the loss of mitochondrial CPT1a and the existence of most β-oxidation proteins bearing a similar PTS to *Tg*ACS1.

Indeed, among the important functions of ACSs, one such function is the channeling of FA toward energy-yielding process that is the β-oxidation (20, 22, 55). The mouse ortholog of human *Hs*ACSBG1, called mBG1, which localizes in the form of vesicles close to neuronal cells’ mitochondria, was shown to be involved in mitochondrial β-oxidation of palmitate, a long-chain fatty acid (25). Interestingly, based on phylogenetic analysis, *Tg*ACS1 clusters together with the *Hs*ACSBG gene family, known to be involved in β-oxidation (20). The presence of FA β-oxidation in Apicomplexa has been relatively unexplored, as the parasite generally uses glycolysis to generate most of its energy. However, initial studies on extracellular stages clearly point to the capacity of the parasite not to rely on exogenous carbon sources to maintain ATP generation and maintain gliding motility (39), and the existence of internal storage to provide such carbon sources for energy supply via oxidative phosphorylation in the mitochondrion, one of them being GABA storage (40). The presence of LD, combined to bioinformatic confirmations for the presence of a complete β-oxidation pathway, and our experimental evidence for the fact that *Tg*ACS1 is a bubble gum ACS that enables parasite gliding motility, together strongly advocate for the existence of β-oxidation in the parasite. In eukaryotes, this energy-yielding process compartmentalizes to mitochondria and vesicular organelles called peroxisomes. Initial reports claimed for the absence of such peroxisomes in *T. gondii*, notably due to unclear localizations and biochemical analyses, as well as the initial bioinformatic absence of PEX proteins, which are key proteins for the organelle biogenesis in other eukaryotes. However, there are now clear genomic and bioinformatic evidence for the presence of peroxisomal-like structure/aggregates/area in *T. gondii* where other proteins bearing a predicted PTS would gather to perform their functions (33, 34). Proteins imported into the peroxisomal lumen bear two canonical targeting sequences: either the PTS1, a C-terminal tripeptide with the consensus sequence [SAC]-[KRH]-[LM], or the PTS2, an N-terminal peptide comprised of the amino acids [RK]-[LVIQ]-X-X-[LVIHQ]-[LSGAK]-X-[HQ]-[LAF] (37). Moog et al. provides bioinformatic evidence for the presence of classical PTSs in protein factors potentially involved in β-oxidation in apicomplexan parasites. The identification of PEX proteins and the experimental evidence for the interaction of the toxoplasma PTS1 with TgPEX5 (37, 38), combined to experimental evidence in this study now robustly point at the existence of peroxisomal-like structures or areas in the parasite. However, the lack of clear detection of such a membrane-bound peroxisome combined to divergence we observed in this study both argue for a “non-conventional” nature of such peroxisomal-like structure/patch in the parasite. Indeed, we identified seven putative ACS enzymes found in *T. gondii* parasites, and *Tg*ACS1 was one of the proteins identified within a “high confidence” PTS1-targeted peroxisomal proteome for *T. gondii*. Importantly, the PTS1 sequence of *Tg*ACS1 is key to provide its correct extracellular localization as this protein re-localizes in a peroxisomal-like zone, as shown by co-localization with other proteins bearing a PTS, which could act as positive controls (catalase, PTS). It is also essential to allow the proper function of *Tg*ACS1 as confirmed by heterologous complementation. The conserved *Toxoplasma* PTS1 sequence was previously demonstrated as being enough and sufficient to efficiently import proteins toward the peroxisome in yeast, thus showing its functionality. PEX5 can recognize and bind to the proteins bearing a PTS1, and which are usually proteins of the matrix (inside/lumen) of the peroxisome. The cargo complex is then imported inside the peroxisome through a pore formed by a complex of other PEX proteins. Hence, this system allows the transport of any soluble protein destined to peroxisomal lumen. Since the interaction of *Tg*PEX5 with PTS1 has previously been shown (38), the systems as actually described are plausible with a putative recognition of *Tg*ACS1 and its PTS1 by *Tg*PEX5 for a relocation somewhere else, whether there is a membrane-bound peroxisome or none. However, unlike typical PTS1-based import in peroxisome, here, the PTS1 could be found active when placed before or after the HA tag at the C-terminal of the protein. It therefore seems unlikely to be mediated by a typical peroxisomal translocation system. Furthermore, the ability to degrade the protein via the AID system suggests that this structure could be membrane-less. One hypothesis is that this structure could rather be like a biomolecular condensate or droplet. Such structures have recently been discovered with major implications in cellular biology: the LLPS (56). Such membrane-less structures form crucial functional structures such as the nucleolus and Cajal bodies in the nucleus, stress granules, and p granules in the cytosol of eukaryotic cells, and even in SARS-COVID virus. This could explain the lack of detection of such membrane-bound peroxisomes throughout the years of cellular scrutiny in *Toxoplasma*. Immuno-electron microscopy of extracellular parasites under nutrient-stress conditions could further determine the presence or absence of potential peroxisomal-like LLPS or compartments, which were previously unconfirmed.

**Fig 9:**
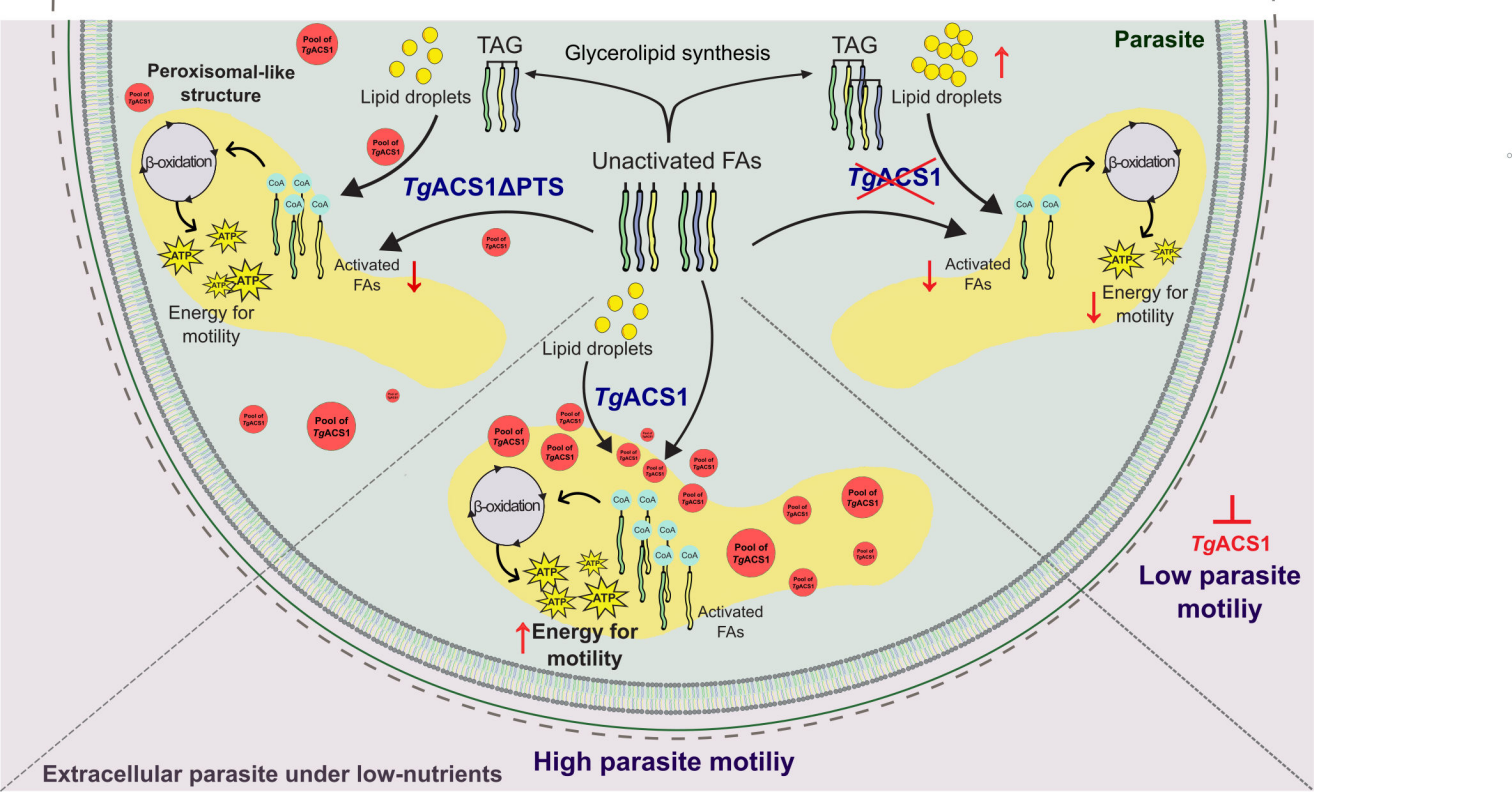
Model for the proposed role of *Tg*ACS1 in extracellular parasites: metabolic activation of fatty acids through PTS1-based re-localization in peroxisomal-like patches allowing parasite motility by putatively fueling parasite β-oxidation pathway. Inactivated FAs from the extracellular environment, the host, and/or *de novo* made by the parasite are incorporated into the glycerolipid pathway of the parasite to form TAGs, which are stored in parasite lipid droplets. When *Tg*ACS1 is present with its predicted PTS1 sequence, the protein relocates in peroxisomal-like structure/patch where it likely fuels the parasite β-oxidation to provide energy and allow parasite motility by activating parasite fatty acids from any source (central part of the diagram). Upon the depletion of *Tg*ACS1 in extracellular parasites, parasite lipid droplet levels increase, while there is a significant decrease in FFA levels, leading to a significant reduction in parasite motility (right side of the diagram). When *Tg*ACS1 is lacking its PTS1 (*Tg*ACS1ΔPTS), there is a milder increase in LD number than without *Tg*ACS1. The pool of *Tg*ACS1 lacking its PTS is found scattered throughout the parasite’s cytosol, preventing the protein to activate FA in bulk at peroxisomal-like patches.

Here, we generated the first lipidome of extracellular parasites and parasites with differing host nutrient conditions. The consistent increase in TAG and decrease in FFA levels under the depletion of *Tg*ACS1 in both intracellular and extracellular parasites, specifically in low nutrient conditions (0%–1% FBS), highlight the nutrient-dependent role of this protein in the mobilization of TAG into activated FFAs (Fig. 9). FFAs can be hydrolyzed from TAG molecules via phospholipases. Therefore, we suspect that ACSs are responsible for the activation and mobilization of these recycled FFAs for other metabolic process, most likely membrane biogenesis and energy support. The relationship between the generation of energy by the parasite and the existence of an active β-oxidation process remains to be further demonstrated, potentially via more sensitive metabolomic approaches than existing ones. Yet, our data points to a correlation between a requirement of FA trafficked through the action of *Tg*ACS1 to a structure/area (i.e., peroxisome-like spots), which is putatively bearing the β-oxidation metabolic function, and cellular processes for which the parasite requires a boost of energy (i.e., motility, in a low nutrient environment). Our data thus now suggest that, in sub-optimal conditions, the parasite is still able to produce energy to facilitate gliding and that *Tg*ACS1 may use glucose as carbon source for gliding motility at the same time. Together, this suggests that *T. gondii* can use energy from lipid catabolism to produce its own energy from its own reserves without the host (Fig. 9). The predicted PTS1 at the C-terminus of *Tg*ACS1 further strengthens its putative role in the elusive β-oxidation pathway in *T. gondii,* and a peroxisome-like area/structure could be the place where FAs are processed for β-oxidation in the parasite. The decrease in motility under low nutrient conditions additionally supports *Tg*ACS1 being involved in the utilization/mobilization of TAGs for energy production in non-ideal conditions. Recent findings in a putative ACSBG protein like *Tg*ACS1 in the microalgae *Microchloropsis gaditana* show a similar increase in TAG levels when the protein is disrupted and pushed toward a role in TAG utilization and β-oxidation (34). It has been reported that one of the sources of energy for the *T. gondii* motility is GABA (40). In our study, there was always some glutamine included, which could be used to fuel the TCA cycle directly or via GABA synthesis. In comparison to the ultimate nutrient deficient condition, i.e., PBS (Fig. S3), the parasite probably still had very low motility in DMEM medium without glucose or FBS. Here, *Tg*ACS1 in the presence FBS showed by the highest increase of motility, suggesting that the *Tg*ACS1-dependent activation of FAs, likely fuelling β-oxidation is a major supply of energy for parasite motility.

We already know from previous work that nutrient availability (9, 19) governs parasite survival. This work brings insight to the parasites’ ability to metabolically adapt to changing host environments and in-depth understanding of the diverse roles of *Tg*ACSs in this process to allow for parasite propagation.

## MATERIALS AND METHODS

### Protein sequence analysis and phylogeny: identification of *Tg*ACSs

Candidate members of the ACS gene family from *T. gondii* were identified using various online bioinformatic tools: Clustal Omega (https://www.ebi.ac.uk/Tools/msa/clustalo/), (https://www.uniprot.org), EuPathDB (https://eupathdb.org/eupathdb). A phylogenetic tree of ACS proteins in several eukaryotes including Apicomplexa, humans, and plants was created using the online platform Phylogeny.fr. The organisms used for ACS protein sequences for generation of the phylogenetic tree include *T. gondii_Tg*ACS1 (TGGT1_297220), *T. gondii_Tg*ACS2 (TGGT1_310150), *T. gondii_Tg*ACS3 (TGGT1_310080), *T. gondii_Tg*ACS4 (TGGT1_243800), *T. gondii_Tg*ACS5 (TGGT1_247760), *T. gondii_Tg*ACS6 (TGGT1_232580), *T. gondii_Tg*ACS7 (TGGT1_276155), *P. falciparum_Pf*ACS1a (PF3D7_1479000), *P. falciparum_Pf*ACS2 (PF3D7_0301000), *P. falciparum_Pf*ACS3 (PF3D7_1253400), *P. falciparum_Pf*ACS4 (PF3D7_1372400), *P. falciparum_Pf*ACS5 (PF3D7_0731600), *P. falciparum_Pf*ACS6 (PF3D7_0401900), *P. falciparum_Pf*ACS7 (PF3D7_1200700), *P. falciparum_Pf*ACS8 (PF3D7_0215300), *P. falciparum_Pf*ACS9 (PF3D7_0215000), *P. falciparum_Pf*ACS10 (PF3D7_0525100), *P. falciparum_Pf*ACS11 (PF3D7_1238800), *P. falciparum_Pf*ACS12 (PF3D7_0619500), *Arabidopsis thaliana*_LACS1 (At2g47240), *A. thaliana*_LACS2 (At1g49430), *A. thaliana*_LACS3 (At1g57920), *A. thaliana*_LACS4 (At4g23850), *A. thaliana*_LACS5 (At4g11030), *A. thaliana*_LACS6 (At3g05970), *A. thaliana*_LACS7 (At5g27600), *A. thaliana*_LACS8 (At2g04350), *A. thaliana*_LACS9 (At1g77590); *Homo sapiens_*ACSS1 (NX_Q9NUB1), *H. sapiens_*ACSS2 (NX_Q9NR19), *H. sapiens_*ACSS3 (NX_Q9H6R3), *H. sapiens_*ACSVL1 (NX_O14975), *H. sapiens_*ACSVL2 (NX_Q9Y2P4), *H. sapiens_*ACSVL3 (NX_Q5K4L6), *H. sapiens_*ACSVL4 (NX_Q6P1M0), *H. sapiens_*ACSVL5 (NX_Q6PCB7), *H. sapiens_*ACSVL6 (NX_Q9Y2P5), *H. sapiens_*ACSL1 (NX_P33121), *H. sapiens_*ACSL3 (NX_O95573), *H. sapiens_*ACSL4 (NX_O60488), *H. sapiens_*ACSL5 (NX_Q9ULC5), *H. sapiens_*ACSL6 (NX_Q9UKU0), *H. sapiens_*ACSS3 (NX_Q9H6R3), *H. sapiens_*ACSM3 (NX_Q53FZ2), *H. sapiens_*ACSM2B (NX_Q68CK6), *H. sapiens_*ACSM1 (NX_Q08AH1), *H. sapiens_*ACSM4 (NX_P0C7M7), *H. sapiens_*ACSM5 (NX_Q6NUN0), *H. sapiens* ACSM6_(NX_Q6P461), *H. sapiens_*ACSBG1 (NX_Q96GR2), *H. sapiens_*ACSBG2 (NX_Q5FVE4); *Chromera velia* (Cvel_26948), *Chromera velia* (Cvel_29920), *C. velia* (Cve1_20092), *C. velia* (Cvel_8845), *C. velia* (Cvel_24841), *C. velia* (Cvel_29002), *C. velia* (Cvel_9659), *C. velia* (Cvel_3410), *C. velia* (Cvel_2306), *C. velia* (Cvel_26948) ; *Cryptosporidium parvum* (cgd5_3200), *C. parvum* (cdg3_2870), *C. parvum* (cdg3_2870) *C. parvum* (cdg3_640) ; *Hammondia hammondi* (HHA_310150), *H. hammondi* (HHA_310080), *H. hammondi* (HHA_276155), *H. hammondi* (HHA_232580), *H. hammondi* (HHA_297220), *H. hammondi* (HHA_243800), *H. hammondi* (HHA_247760); *Neospora caninum* (NCLIV_063970), *N. caninum* (NCLIV_006300), *N. caninum* (NCLIV_006990), *N. caninum* (NCLIV_054250), and *N. caninum* (NCLIV_018500). First step has required the initial curation of these protein sequences. The protein sequences were aligned (Clustal Omega) and then gaps were removed from the alignment. Finally, the phylogenetic tree was constructed using the maximum likelihood method in the PhyML program. The default substitution model of the PhyML software was chosen (the so-called “WAG” model) was selected. Graphical representation and edition of the phylogenetic tree were performed with cladogram.

### *T. gondii* strains and cultures

The parasite host cells human foreskin fibroblasts (HFF) were cultured using DMEM (Gibco) supplemented with 10% FBS (Gibco), 2 mM glutamine (Gibco), and 25 µg/mL gentamicin (Gibco) at 37°C and 5% CO_2_. *T. gondii* tachyzoite parental strains RH-ΔKu80 TIR as well as the mutant strain were cultured by serial passage within their host HFF using DMEM supplemented with 1% FBS (Gibco), 2 mM glutamine (Gibco), and 25 µg/mL gentamicin (Gibco) at 37°C and 5% CO_2_.

### Generation of inducible knockdown line for *Tg*ACS1

A C-terminally tagged HA line with inducible post-translational protein degradation was generated using the AID system. A 2,685 bp homology region of the C-terminal end of *Tg*ACS1 excluding the stop codon was amplified using the primers in Table 1. A guide sequence (Table 1) was selected near the C-terminal end and was less than 100 bp away from at least one homology region. The guide sequence was inserted into Cas9 U6 universal plasmid Q5 mutagenesis.

**TABLE 1:** Primers list used in this study

*Tg*ACS gene ID	Primers (gene-specific sequence underlined)	Molecular system used
*Tg*ACS1 (TGGT1_297220)	Fw: GCAGCATGAGAAAACCGTTGCTGCGCTGCAGGCAAAGCTGGCTAGCAAGGGCTCGGGC Rw: CCTTGACCGCCGCAGAGATGTTCTTCTCCCTTTTGCCAGGATAGGGCGAATTGGAGCTCC Rw2: CGCGTCCCCGCAGCATGAGAAAACCGTTGCTGCGCTGCAGGCTAGCAAGGGCTCGGGC Guide sequence: CGAATTTCAGAAGCTGCCGA SS55: CGCTCTCCCCTGCCTTCTTG SS48: ATACATTCGCAAATCAATCTTTCGCAGGTACGGC SS49: TTGAAGTCGTAGCAGCAACCAACGA SS56: CGCACAACAAGGAAATGTGATGAAGG	mAID
*Tg*ACS2 (TGGT1_310150)	Fw: TGTTCCAGATTATGCCTTACCCGGGATGCCTGTCTCGGGCGCT Rw: TGGAGCTCCACCGCGGTGGCGCGGCCGCCGACCACCACGACCGCAG	TATI-Tet off
*Tg*ACS1 (TGGT1_297220)	Fw: TACTTCCAATCCAATTTAATGCAGCCTGGGGATGCCGATCAACT	pLIC HA tagging
	Rw: TCCTCCACTTCCAATTTTAGCCAGCTTTGCCTGCAGCGC	
*Tg*ACS2 (TGGT1_310150)	Fw: TACTTCCAATCCAATTTAATGCGCGCTCGGCGTTGAGTT Rw: TCCTCCACTTCCAATTTTAGCCGACCACCACGACCGCA	pLIC HA tagging
*Tg*ACS3 (TGGT1_310080)	Fw: TACTTCCAATCCAATTTAATGCACTTGGCCATCTCCGCGTATCC Rw: TCCTCCACTTCCAATTTTAGCCACGCTGTGGCTGAGTTCGTC	pLIC HA tagging
*Tg*ACS4 (TGGT1_243800)	Fw: TACTTCCAATCCAATTTAATGCCTTGCTTGGTGGCCATCATCG Rw: TCCTCCACTTCCAATTTTAGCAATCGCCTTCGCTCTCTCCG	pLIC HA tagging
*Tg*ACS5 (TGGT1_247760)	Fw: TACTTCCAATCCAATTTAATGCCCTCGGATCATCGACCGAGC Rw: TCCTCCACTTCCAATTTTAGCATTGGCAGGATGGTGCCGT	pLIC HA tagging
*Tg*ACS6 (TGGT1_232580)	Fw: TACTTCCAATCCAATTTAATGCGAAGATGAGATGACCGGGC Rw: TCCTCCACTTCCAATTTTAGCCTTATCTTCGACGTCCTTTACAG	pLIC HA tagging
*Tg*ACS7 (TGGT1_276155)	Fw: TACTTCCAATCCAATTTAATGCGAAGCAGGCGAACCTGGA Rw: TCCTCCACTTCCAATTTTAGCGTCGTCGTTCTTCAAAAGTTCG	pLIC HA tagging
*Tg*ACS1 (TGGT1_297220)	Fw: TCCCTTTCGTCATGAAACTCA Rw: GCCGTAAATGGAGTTGATCG	mRNA relative expression

The PCR product and the Cas-9 guide plasmid were used for transfection of a RH TIR1-3FLAG parental line (47). After 24 h, the newly transfected line was treated with 25 µg/mL mycophenolic acid and 50 µg/mL xanthine. Parasites were selected with the drug and cloned by limiting dilution. Screening of parasite clones for correct insertion of the mAID sequence, Hypoxanthine-Xanthine (HX) drug cassette and overall insertion/orientation were done with the primers in Table 1. All PCRs were performed with TaKara Primestar Max polymerase. The knockdown of *Tg*ACS1 was induced with a final concentration of 100 µM of indole-3-acetic acid (auxin) made up in 100% ethanol.

A similar mutant line was also generated using the RH-TIR-3FLAG parental line that excluded the PTS tripeptide sequence. This was done using the same method described above but instead making use of the Fw primer and Rw2 primer (Table 1) to generate the PCR product (including the homology regions at each end of the cut site, the mAID sequence, and drug cassette and excluding the PTS) that was then inserted into the genome of the parasite during transfection (47). All PCRs were performed with TaKara Primestar Max polymerase.

### Immunofluorescence assay

Primary antibodies anti-HA (Rat, Roche), anti-Inner Membrane Complex protein1 (IMC1), anti-SAG1, anti-proM2AP, and anti-AMA1 were used at dilutions 1:500, 1:1,000, 1:1,000, 1:1,000, and 1:1,000, respectively. Secondary Alexa Fluor 488- and 546-conjugated anti-mouse, anti-rat, and anti-rabbit antibodies (Life Technologies) were used at 1/2,500. For the IFA, parasites were grown on confluent HFF on coverslips and fixed in PBS containing 2.5% paraformaldehyde (PFA) for 15 min at room temperature (RT). Samples were permeabilized with 0.25% Triton X-100 in PBS for 10 min at RT prior to blocking in PBS containing 3% Bovine Serum Albumine (BSA) and subsequent incubation with primary antibodies then secondary antibodies diluted in the blocking solution. Labeled parasites were stained with Hoechst (1/10,000, Life Technologies) for 20 min and then washed three times in PBS before final mounting of the coverslips on a glass slide using fluorogel. The fluorescence was visualized using fluorescence microscope (Axio Imager 2_apotome; ZEISS).

### Nile red staining of lipid droplets

Parasites were allowed to infect and grow in confluent monolayer HFF grown on coverslips, in the +/−ATc or auxin conditions for 24 h in 0%/1%/10% FBS-containing culture medium medium. As with IFA, these coverslips were fixed using 2.5% PFA, permeabilized with 0.25% Triton X-100. Thereafter, coverslips were incubated for at least 1 h with Nile red (or HCS LipidTOX, 1:200) in 1× PBS before proceeding to DNA staining with Hoechst. The coverslips were mounted onto a glass slide in fluorogel prior to imaging using fluorescence microscope (Axio Imager 2_apotome; ZEISS). For visualizing Nile red-stained droplets, yellow-gold fluorescence (excitation, 450–500 nm; emission, greater than 528 nm) was used on the Axio Imager. Quantification of lipid droplet number and volume was preformed based on iterative image processing as described by Exner et al. (57).

### Western blot analysis

Parasites were harvested for Western blot after complete egress from their host. In order to remove any host cell debris, the parasites were passed through a 3 µm filter, then counted by hemocytometer and solubilized in SDS buffer at equivalent cell densities. Equal amount of protein was separated on a 4%–12% gradient SDS-polyacrylamide (Life Technologies) and transferred to nitrocellulose membrane (check this) using the XCell II Blot Module (Invitrogen). Primary antibodies anti-HA (rat, Roche) and anti-TOM40 (rabbit) (58) were used at a dilution of 1:500 and 1:1,000, respectively. Secondary goat anti-mouse and anti-rabbit horse radish peroxidase (HRP)-conjugated antibodies (Thermo Scientific) were used at 1:2,000. Protein signal was detected by chemiluminescence after membrane staining with Luminata Crescendo Western HRP detection kit (Millipore). The signal strength of protein was quantified using a BioRad Chemidoc imager (BioRad).

### Phenotypic analysis

#### Plaque assay

The extracellular parasites were harvested after filtration and counted by hemocytometer. Then, approximately 500 parasites were inoculated to confluent HFF flask (25 cm^2^). The mutant parasites *Tg*ACS1-iKD and *Tg*AC2-iKD were grown for plaque assay in the presence or absence of ATc or auxin (0.5 µg mL−1 or 100 µM) for 7–10 days. Plaque sizes were visualized by crystal violet staining (30–60 min) after aspiration of culture media, and cell fixation with 100% ethanol (5 min) followed by PBS wash.

#### Replication assay

The parasites were grown for 2 days with or without ATc or auxin, harvested, and filtered. Equal number of parasites were allowed to invade confluent HFF grown on coverslips. Following 2 h of invasion, the coverslips were washed thrice with ED1 (1% FBS containing DMEM), in order to remove any uninvaded parasites and promote synchronized replication. ATc or auxin (same concentration as above) was added at the outset of the experiment, allowing the treatment for 24 h, alongside control parasites without auxin. These coverslips were then fixed and processed for IFA wherein the parasite number per parasitophorous vacuole was analyzed.

#### Motility assay

Slides were prepared before start of the experiment, whereby 1.5 mm glass slides were flamed and smeared with 0.1% polyethyleamine. Slides were placed in six-well plates and incubated with a layer of FBS for 2–3 h at 37°C. Freshly egressed *Tg*ACS1 iKD parasites were passed through a 3 µM filter and spun down for 10 min at 1,800 rpm. Cells were washed with PBS three times and resuspended in 1 mL of ED1 media (100 mM HEPES) or PBS (100 mM HEPES), in conditions with or without 100 mM auxin. Before placing the cell suspension, slides were washed three times with PBS to remove excess FBS on the glass slides. Cells where incubated within the wells for 10 min at room temperature to allow cells to settle and for another 1 h at 37°C. Samples were fixed with 5% paraformaldehyde/PBS for 15 min and processed for an IFA using anti-SAG1 antibody. The experiment was performed three independent times.

#### Relative mRNA expression quantification

RH TIR1-3FLAG parasites were grown on confluent monolayer of HFF in flasks (175 cm^2^) and allowed to grow for 48 h (mainly intracellular) or until completely egressed (extracellular parasite) in 0%, 1%, or 10% FBS normal DMEM conditions. Parasites were then harvested by scraping cells at the bottom of the flask and moved to 50 mL tubes; intracellular tachyzoites were pushed through a 0.2 mm needle and syringe filtration with 3 µm pore size membrane to remove host cell debris. A NucleoSpin RNA kit (Macherey-Nagle) was used for mRNA extraction, and whereafter, cDNA was synthesized using a SuperScript III First-Strand Synthesis SuperMix for qRT-PCR kit. Primers specific (Table 1) to each gene of interest were designed and used to determine mRNA expression, including a housekeeping gene GAPDH1 (TGGT1_289690), which was used to calculate the relative mRNA expression in the different conditions used in this study.

### Lipidomic analysis

The parasites were grown for 24 h or until completely extracellular in conditions of +/−auxin on the confluent monolayer of HFF in flasks (175 cm^2^). For extracellular parasite lipid analyses, fully egressed parasites were collected, then, host cells were filtered out with a with 3 µm pore size membrane, after which, parasites were incubated for 6 h in the respective media conditions (0%/1%/10% FBS). At each time point, parasites were also harvested as the intracellular tachyzoites (1 × 107 cell equivalents per replicate) after syringe filtration with 3 µm pore size membrane. These parasites were metabolically quenched by rapid chilling in a dry ice-ethanol slurry bath and then centrifuged down at 4°C. The parasite pellet thus obtained was washed with ice-cold PBS thrice, before transferring the final pellet to a microcentrifuge tube. Then, total lipids were extracted in chloroform/methanol/water (1:3:1, vol/vol/vol) containing Phosphatidylcholine (PC) (C13:0/C13:0), 10 nmol, and C21:0 (10 nmol) as internal standards for extraction) for 1 h at 4°C, with periodic sonication. Then, polar and apolar metabolites were separated by phase partitioning by adding chloroform and water to give the ratio of chloroform/methanol/water, 2:1:0.8 (vol/vol/vol). For lipid analysis, the organic phase was dried under N_2_ gas and dissolved in 1-butanol to obtain 1 µl butanol/10^7^ parasites.

#### Total lipid analysis

Total lipid was then added with 1 nmol pentadecanoic acid (C15:0) as internal standard and derivatized to give fatty acid methyl ester (FAME) using trimethylsulfonium hydroxide (Macherey-Nagel) for total glycerolipid content. Resultant FAMEs were then analyzed by GC-MS as previously described (59). All FAMEs were identified by comparison of retention time and mass spectra from GC-MS with authentic chemical standards. The concentration of FAMEs was quantified after initial normalization to different internal standards and finally to parasite number.

#### Phospholipid and neutral lipid analysis

For phospholipid analysis, total lipid extracted (as mentioned above) was separated with 1 nmol PA (C17:0/C17:0) (Avanti Polar lipids) by one-dimensional silica gel HPTLC (Merck). The solvent system used for the first and second dimension was chloroform/methanol/28% ammonium hydroxide, 12:7:1.6 (vol/vol), and chloroform/acetone/methanol/acetic acid/water, 10:4:2:2.6:1 (vol/vol/vol/vol/vol), respectively. Total fractions of PL, DAG, TAG, FFAs, and cholesteryl ester obtained from total parasite lipid fraction using 1D HPTLC separation in hexane/diethyl ether/formic acid, 80:20:2 (vol/vol/vol) as solvent system. Thereafter, each lipid spot on the HPTLC plate was scrapped off and lipids were methanolized with 200 µL 0.5 M methanolic HCl in the presence of 1 nmol pentadecanoic acid (C15:0) as internal standard at 85°C for 3 h. The resulting FAMEs were extracted with hexane and finally analyzed by GC-MS (Agilent).

### Yeast complementation assays and growth curves

A pRS426-MET25 vector was used as the backbone for a plasmid containing the entire cDNA sequence of *Tg*ACS1. The plasmid was synthesized (GeneCust) by cloning the predicted transcribed sequence from *Tg*ACS1 (which was obtained from Toxodb.org, and codon optimized for heterologous expression in yeasts) using *Eco*RI and *Kpn*I restriction sites. All strains were transfected either with this plasmid of the pRS426-MET25 vector lacking *Tg*ACS1 as a control.

The isogenic *S. cerevisiae* strains used in this study (i.e. WT: YB332: MATa ura3 leu2 his3A200 ade2 lys2-801, and *faa1*∆*faa4*∆: YB525: MATa ura3 leu2 his3D200 ade2 lys2–801 faa1∆::HIS3; faa4∆::LYS2) were kindly provided by Dr. Juliette Jouhet [Commissariat aux Energies Atomiques et aux Energies Alternatives (CEA), Grenoble, France] and have been described earlier (50, 51). After transfection, all the strain of the present study were grown on synthetic medium containing 6.7 g/L of YNB (MP Biomedicals), 0.77 g/L of CSM-URA (i.e. Complete Supplement Mixture-Uracil, MP Biomedicals) and g/L of glucose, as described. In complementation experiments, the media were supplemented with 22.5 mM cerulenin (Sigma-Aldrich), 100 mM FFA, and 0.2% (vol/vol) final concentration of Tween 20 (Sigma-Aldrich). The different FFAs were dissolved in 100% Tween 20 at a concentration of 50 mM. Cerulenin was dissolved in acetone at 45 mM. As controls, media without FFAs and/or cerulenin were also supplemented with 0.2% (vol/vol) Tween 20 and/or acetone.

The isogenic *S. cerevisiae* strains used in this study (i.e. WT (YB332: MATa ura3 leu2 his3A200 ade2 lys2-801) and *faa1*∆*faa4*∆ (YB525: MATa ura3 leu2 his3D200 ade2 lys2–801 faa1∆::HIS3; faa4∆::LYS2) were kindly provided by Dr. Juliette Jouhet (Commissariat aux Energies Atomiques et aux Energies Alternatives (CEA), Grenoble, France) and have been described earlier (52, 53). After transfection, all the strains described in the present work were grown on synthetic medium containing 6.7 g/L of YNB (MP Biomedicals), 0.77 g/L of CSM-URA (MP Biomedicals) and g/L of glucose, as described. In complementation experiments, the media were supplemented with 22.5 mM cerulenin (Sigma-Aldrich), 100 mM FFA, and 0.2% (vol/vol) final concentration of Tween 20 (Sigma-Aldrich). The different FFAs were dissolved in 100% Tween 20 at a concentration of 50 mM. Cerulenin was dissolved in acetone at 45 mM. As controls, media without FFAs and/or cerulenin were also supplemented with 0.2% (vol/vol) Tween 20 and/or acetone.

Three clones per transformation were used for complementation tests. For each experiment, one colony was inoculated in 6 mL of CSM-URA + glucose (2%) and grown overnight at 30°C at 180 rpm. Growth assays were performed in 96-well microplates (Thermo Scientific Nunc MicroWell 96-Well, Nunclon Delta-treated, Flat-Bottom Microplate) filled with 200 mL of culture in glucose medium supplemented or not by FA or cerulenin and set at an OD600: optical Density (OD_600_= 0.1). The microplate was incubated at 30°C in an Infinite M2000 PRO microplate reader (Tecan) set for plate orbital shake for 10 s and absorbance measurements (OD_600_) every 20 min, with four measurements per well. OD_600_ readings from experimental wells minus the original OD_600_ values resulted in the ∆OD_600_ values shown in Fig. S5.

We transfected the pRS426-MET25-*Tg*ACS1 plasmid into a yeast strain lacking a peroxisomal-CoA synthetase, *∆*PCS60 (17, 35). These experiments were also performed in Tween 20, with and without 12 µg/mL doxorubicin, a cancer drug inducing lipid peroxidation and peroxisome proliferation in yeast cells (23, 60). OD_600_ was calculated as described above.

### Statistical analysis for all experiments

Entire graphical data for this study were generated using GraphPad prism software. Three biological replicates were used per experiment (*n* = 3). The error bars are representative of standard error of mean for each study. Statistical significance was determined for each experiment by *t*-test using GraphPad Prism (version 8). Range of statistical significance was signified as per the *P*-value, wherein 0.01–0.05 = *, 0.01–0.001 = **, and <0.001 = ***.

## SUPPLEMENTAL MATERIAL

Supplemental material mbio.00427-24-s0001.docx Supplemental figures.

ASM does not own the copyrights to Supplemental Material that may be linked to, or accessed through, an article. The authors have granted ASM a non-exclusive, world-wide license to publish the Supplemental Material files. Please contact the corresponding author directly for reuse.
