# Supplementary material for: The acyl-CoA synthetase TgACS1 allows neutral lipid metabolism and extracellular motility in Toxoplasma gondii through relocation via its peroxisomal targeting sequence (PTS) under low nutrient conditions
Source: mBio. 2024 Mar 19;15(4):e00427-24. doi: 10.1128/mbio.00427-24 (PMC11005404; doi:10.1128/mbio.00427-24)
Supplement: Supplemental material — Supplemental figures. [file mbio.00427-24-s0001.docx]

***The acyl-CoA synthetase TgACS1 allows neutral lipid metabolism and extracellular motility in Toxoplasma gondii through relocation via its peroxisomal targeting sequence (PTS) under low nutrient conditions.***

Sarah Charital^1,+^, Serena Shunmugam^1,+^, Sheena Dass^1^, Anna Maria Alazzi^2^, Christophe-Sébastien Arnold^1^, Nicholas J. Katris^1^, Samuel Duley^1^, Nyamekye A. Quansah^1^, Fabien Pierrel^3^, Jérôme Govin^2^, Yoshiki Yamaryo-Botté^1^*, Cyrille Y. Botté^1^*

1. Apicolipid Team, Institute for Advanced Biosciences, CNRS UMR5309, INSERM U1209, Université Grenoble Alpes, Grenoble, France.
2. Team Govin, Institute for Advanced Biosciences, CNRS UMR5309, INSERM U1209, Université Grenoble Alpes, Grenoble, France.
3. Université Grenoble Alpes, CNRS, Grenoble INP, TIMC-IMAG, 38000 Grenoble, France.

^+^These authors contributed equally

*Equal senior and corresponding authors. To whom correspondence should be [cyrille.botte@univ-grenoble-alpes.fr](mailto:cyrille.botte@univ-grenoble-alpes.fr) ; [yoshiki.botte-yamaryo@univ-grenoble-alpes.fr](mailto:yoshiki.botte-yamaryo@univ-grenoble-alpes.fr)

***Supplementary Figures:***

***
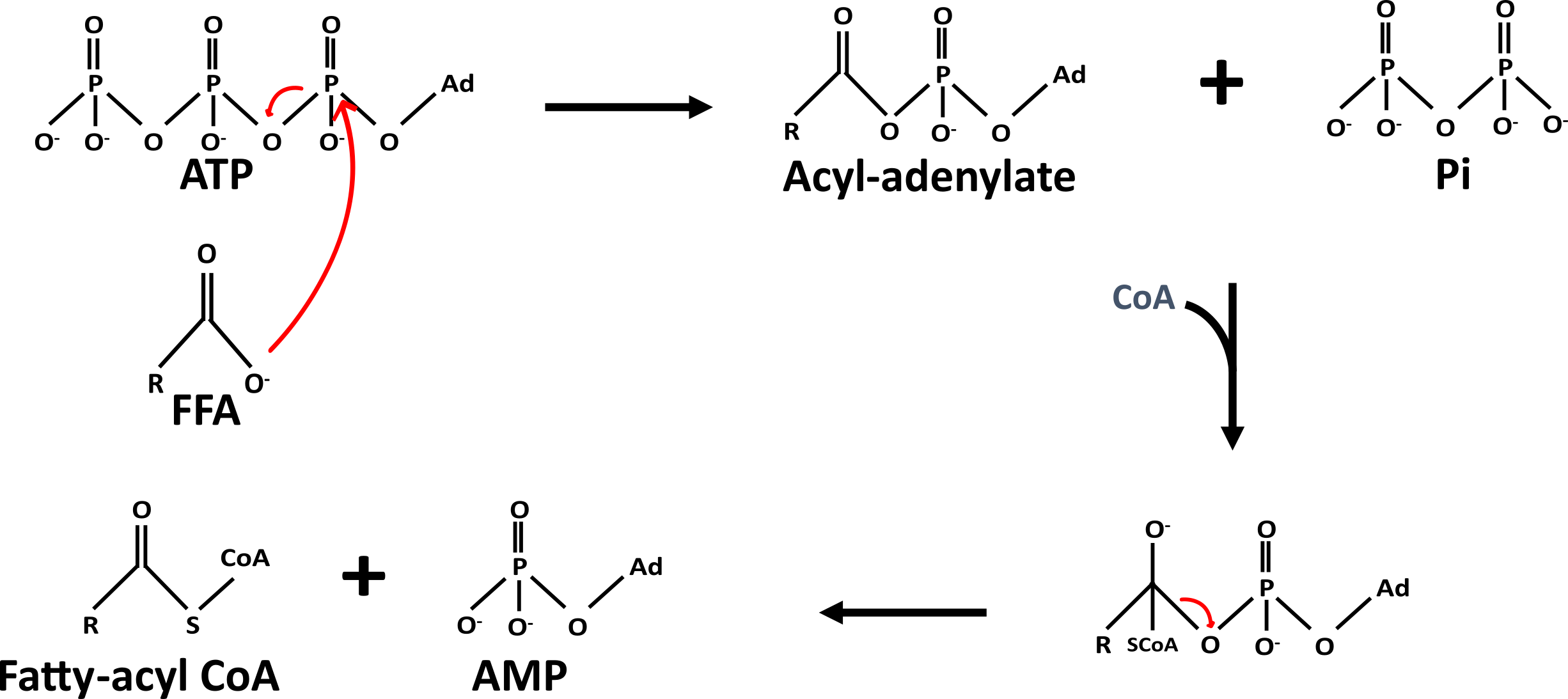
***

**Supplementary Figure 1 related to the introduction.** Acyl CoA synthetases (ACSs) catalyse the activation of free FA by the thioesterificaiton of Coenzyme A (CoA) on the FA moiety. The initial step involves the formation of an adenylated intermediate through the hydrolysis of an ATP molecule, thus releasing pyrophosphate (Pi). The ATP-activated enzyme then binds to the carboxyl group of an incoming free FA (FFA) through an acyl bond to the phosphoryl group of AMP. The final Acyl-CoA product is formed after the transfer of the Acyl group to the sulfhydryl group of coenzyme A, thereby releasing AMP.

**
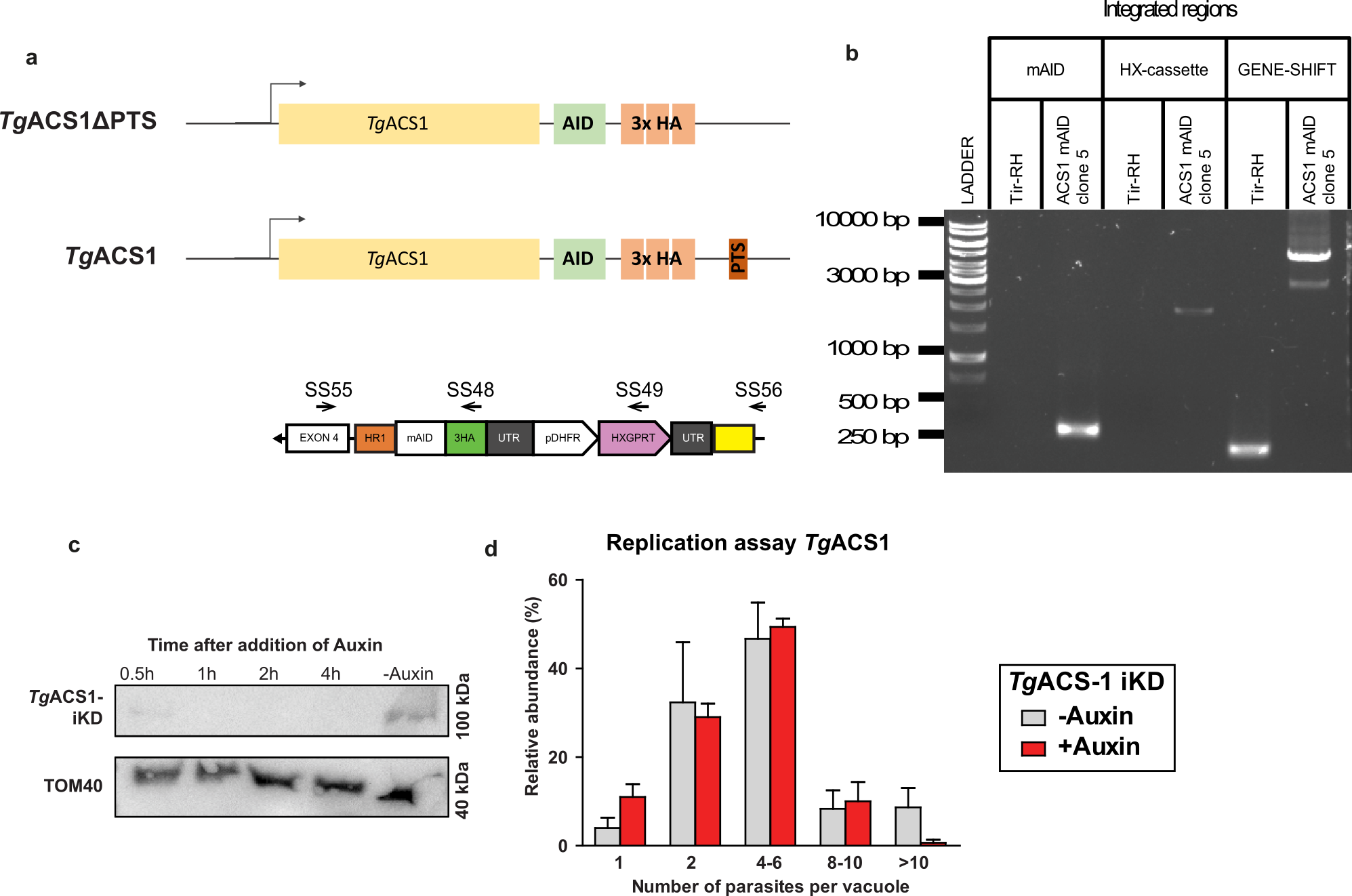
Supplementary Figure 2 related to Figure 3:** a) Scheme representing the constructs used during the study: *Tg*ACS1-mAID-HA-iKD and *Tg*ACS1ΔPTS-mAID-HA-iKD. b) PCR confirmation of *Tg*ACS1-iKD clone 5 using the mAID system, showing correct integration of the mAID sequence, HX-drug cassette, and the gene-shift compared to the parental line. c) Western blot analyse was performed at three different time points of auxin treatment, 30 minutes, 1h, 2h and 4h, and show a down regulation of *Tg*ACS1 (100 kDa) after 30 min of auxin treatment (100 µM), TOM40 (40 kDa) antibody used as loading controls. d) A replication assay of this line with (grey/-Auxin) and without (red/+Auxin) *Tg*ACS1 revealed no effect on intracellular parasite replication. e) The number of lipid droplets per intracellular parasite were not affected at 1% FBS but significantly reduced under complete nutrient loss (PBS), with (grey/-Auxin) and further decreased without (red/+Auxin) the protein of interest.


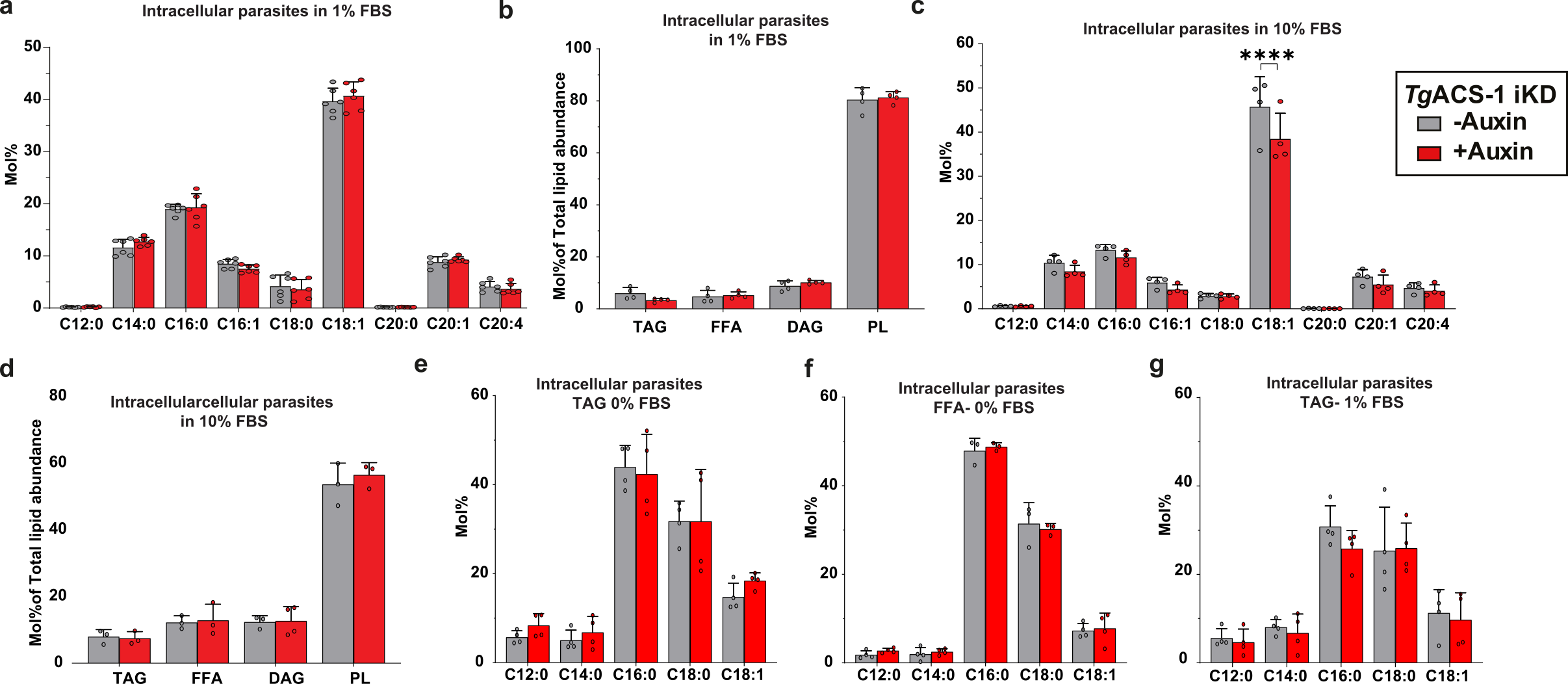


**Supplementary Figure 3 related to figure 4:** Lipidomic profiles showing the mol% of each fatty acid comprising the total lipid fraction of intracellular parasites in a) 1% FBS and c) 10% FBS with (grey/-Auxin) and without (red/+Auxin) *Tg*ACS1. Neutral lipid mol% of total lipid abundance in intracellular parasites in b) 1% FBS and d) 10% FBS with (grey/-Auxin) and without (red/+Auxin) *Tg*ACS1, namely triacylglycerol (TAG), free fatty acid (FFA), diacylglycerol (DAG) and phospholipid (PL). Mol% of e) TAG and f) FFA levels in extracellular parasites at 0% FBS and g) TAG levels in extracellular parasites at 1% FBS.


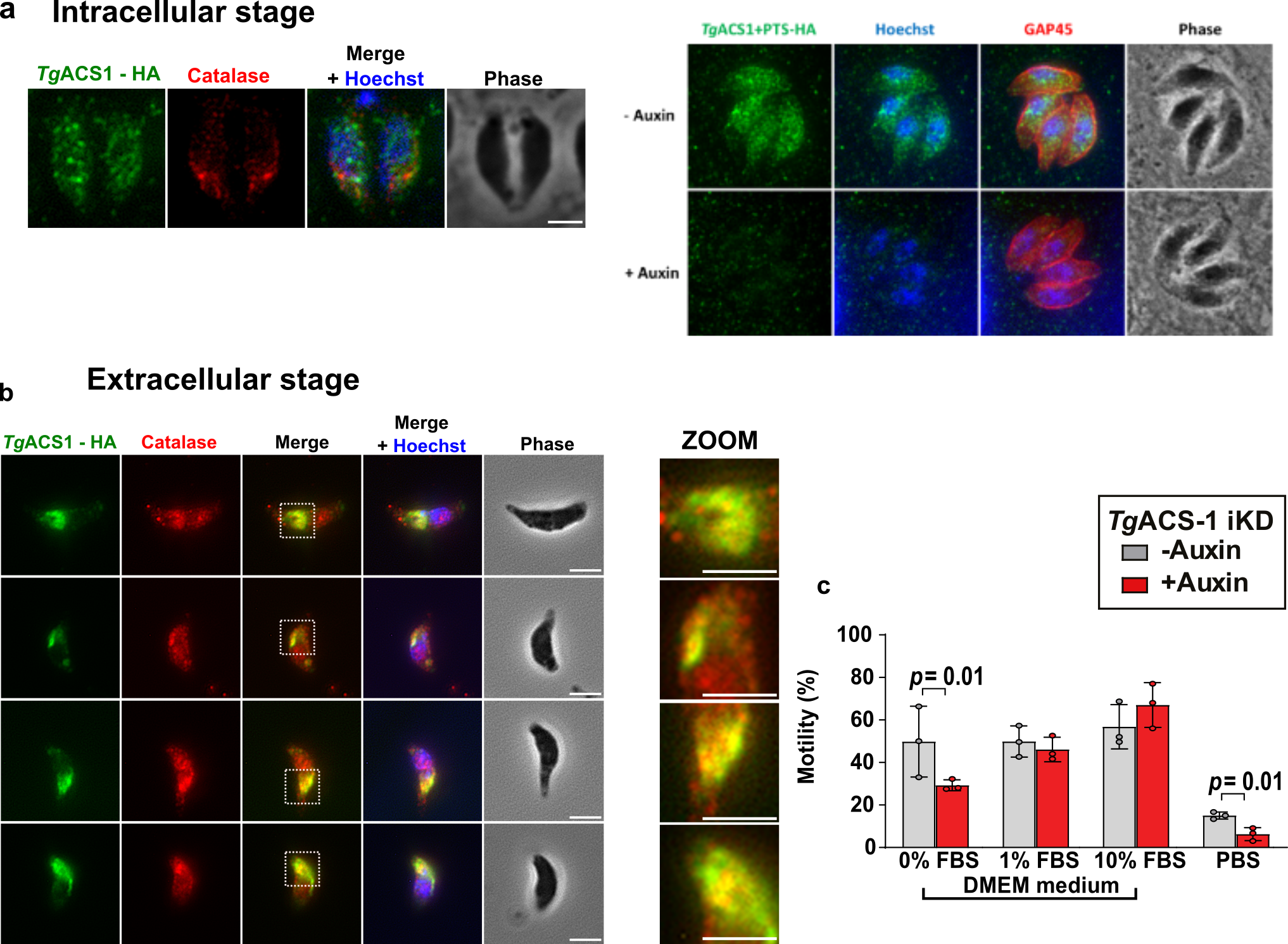


**Supplementary Figure 4 related to figure 5**: a) Immunofluorescence microscopy of intracellular parasites shows no co-localization of *Tg*ACS1, in green, with a peroxisomal marker *Tg*Catalase, in red b) Immunofluorescence microscopy of extracellular parasites revealed *Tg*ACS1 expression and localization in a vesicular anterior pole Golgi-like structure (1% FBS). Co-localisation of *Tg*ACS1 (in green) with a peroxisomal-like markers (anti-TgCatalase, in red) show close proximity to *Tg*ACS1 c) A gliding motility assay showed that under only under complete nutrient loss (PBS, 2 h) and low nutrient conditions like 0% FBS, is the motility rate reduced without *Tg*ACS1 (red/+Auxin), in extracellular proteins.

*
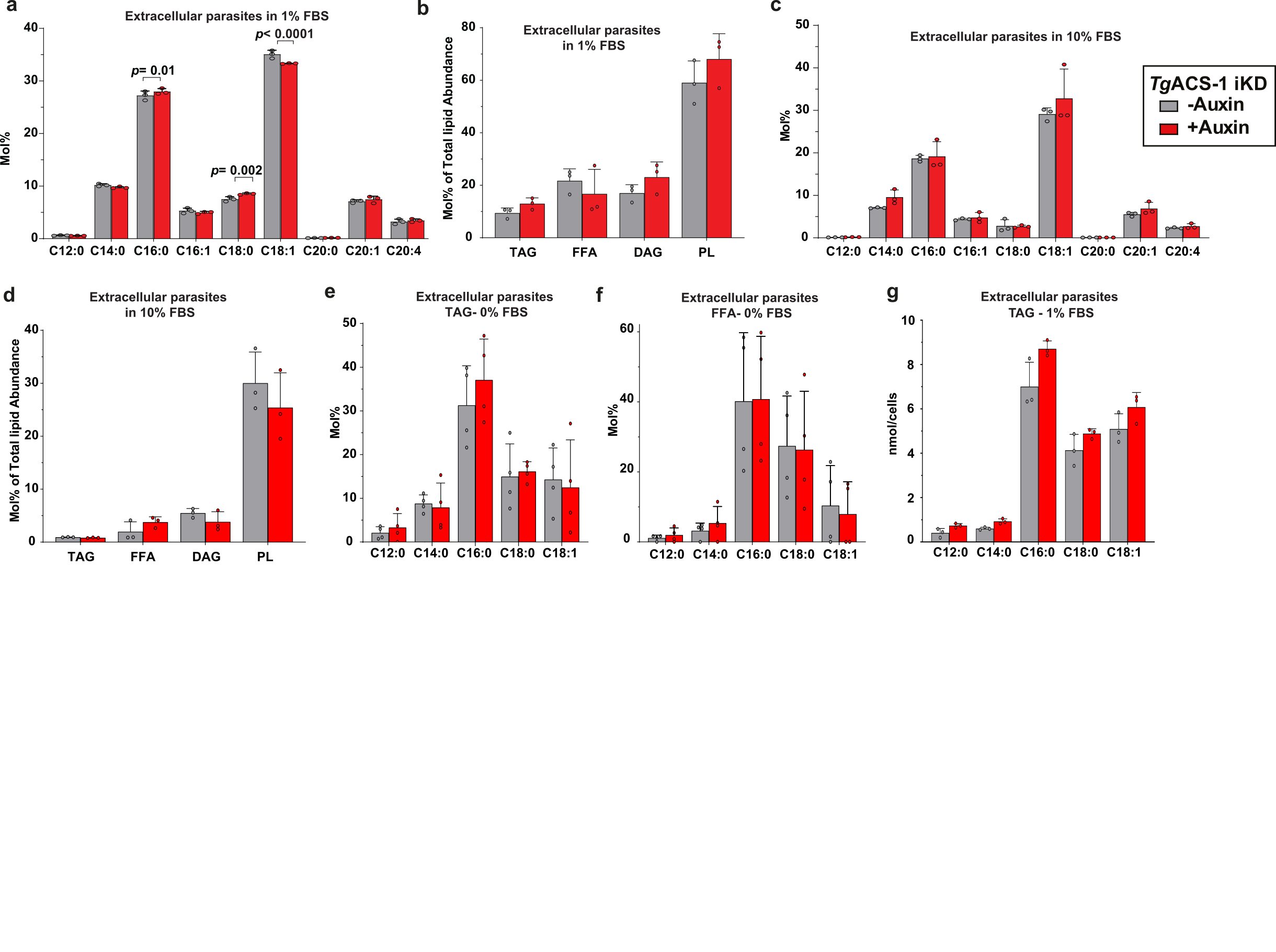
*

**Supplementary Figure 5 related to figure 7:** Lipidomic profiles showing the mol% of each fatty acid comprising the total lipid fraction of extracellular parasites in a) 1% FBS and c) 10% FBS with (grey/-Auxin) and without (red/+Auxin) *Tg*ACS1. Neutral lipid mol% of total lipid abundance in extracellular parasites in b) 1% FBS and d) 10% FBS with (grey/-Auxin) and without (red/+Auxin) *Tg*ACS1, namely triacylglycerol (TAG), free fatty acid (FFA), diacylglycerol (DAG) and phospholipid (PL). Mol% of e) TAG and f) FFA levels in extracellular parasites at 0% FBS and g) TAG levels in extracellular parasites at 1% FBS in nmol/cells.

**
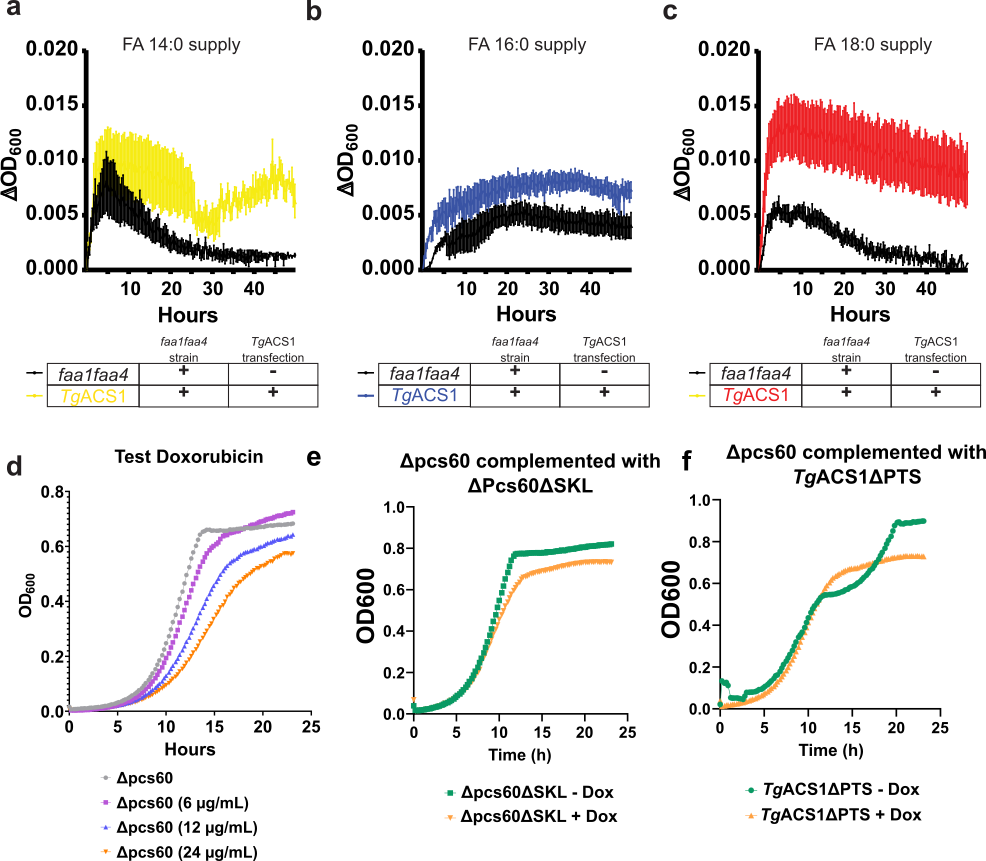
**

**Supplementary Figure 6 related to figure 8:** a-c) Growth curves of the complemented strain under Cerulenin treatment supplied with different FFAs; a) C14:0 (yellow), b) C16:0 (blue) or c) C18:0 (red), with their negative controls of *faa1∆faa4∆* double mutant without complementation in the presence of the respective FFAs. d) the concentration of doxorubicin treatment was tested at 6, 12 and 24μg/mL on the Δpcs60 yeast strain. e) growth complementation of the yeast mutant strain deficient of peroxisomal acyl-CoA activity (ΔPCS60) complemented with the *Tg*ACS1ΔPTS in the absence (in green) or presence (in orange) of doxorubicin. f) growth complementation of the yeast mutant strain deficient of peroxisomal acyl-CoA activity (ΔPCS60) complemented with the Δpcs60ΔSLKL in the absence (in green) or presence (in orange) of doxorubicin. Both results shows a growth decrease of *Tg*ACS1ΔPTS and Δpcs60ΔSLKL suggesting that the PTS sequence is important to rescue the growth of ΔPCS60 yeast strain. Error bars indicate standard deviation (SD), experiments were conducted in independent triplicates.
